# Fine-tuning sugar content in strawberry

**DOI:** 10.1186/s13059-020-02146-5

**Published:** 2020-09-03

**Authors:** Sinian Xing, Kunling Chen, Haocheng Zhu, Rui Zhang, Huawei Zhang, Bingbing Li, Caixia Gao

**Affiliations:** 1 State Key Laboratory of Plant Cell and Chromosome Engineering, Center for Genome Editing, Institute of Genetics and Developmental Biology, Innovation Academy for Seed Design, Chinese Academy of Sciences, Beijing, China; 2 College of Advanced Agricultural Sciences, University of Chinese Academy of Sciences, Beijing, China; 3 College of Horticulture, China Agricultural University, Beijing, China

**Keywords:** Upstream open reading frame, Fine-tuning, Basic leucine zipper, Quantitative trait variation, Asexually reproducing crops, Strawberry, Sugar content

## Abstract

Fine-tuning quantitative traits for continuous subtle phenotypes is highly advantageous. We engineer the highly conserved upstream open reading frame (uORF) of *FvebZIPs1.1* in strawberry (*Fragaria vesca*), using base editor A3A-PBE. Seven novel alleles are generated. Sugar content of the homozygous T1 mutant lines is 33.9–83.6% higher than that of the wild-type. We also recover a series of transgene-free mutants with 35 novel genotypes containing a continuum of sugar content. All the novel genotypes could be immediately fixed in subsequent generations by asexual reproduction. Genome editing coupled with asexual reproduction offers tremendous opportunities for quantitative trait improvement.

## Background

To meet the demands of the expanding world population and climate change, it is critical to accelerate plant breeding. However, limited sources of the genetic variation underlying quantitative traits pose a challenge to plant breeders [1]. This problem is more severe in asexually reproducing crops, such as strawberry, potato, sugarcane, and grape, as genetic diversity within these species is extremely limited due to the absence of sexual reproduction [2]. However, these species offer one major advantage: once a desirable quantitative genotype is generated, it can be propagated for many generations by asexual reproduction.

In plants, the genetic control of major traits is usually complex, and strong or null alleles frequently have deleterious pleiotropic effects [3–5]. Fine-tuning of quantitative traits so as to balance opposing phenotypic side-effects by generating a series of subtle allelic changes is essential for generating novel alleles that can be harnessed for breeding [6, 7]. Genome editing technology is a powerful approach for creating novel allelic variation to fine-tune quantitative traits [1, 8–10]. For example, in tomato, a wide range of *cis*-regulatory mutations in the *SlCLV3* promoter was created by CRISPR/Cas9 genome editing [1]. Remarkably, the diverse types and strengths of the promoter alleles provided a continuum of fruit size variation. Base editing, in which Cas9 variants fused with cytidine or adenosine deaminases are used to directly generate programmed base changes without requiring DNA double-strand breaks or exogenous DNA template [11–15], is an attractive and powerful tool for creating novel genetic changes in promoters, coding sequences, and upstream open reading frames (uORFs).

uORFs play important regulatory roles in a wide range of biological processes [16–19] and are abundant in eukaryotic genes, being present in about 13% of yeast genes, 35% of *Arabidopsis* genes, and 49% of human genes [16, 20, 21]. While most uORFs are not conserved, some encode small peptides that are significantly conserved [22]. uORFs typically reduce the translation efficiency of the main coding region of an mRNA by altering ribosome activity [16]. Engineering uORFs has great potential for modifying metabolic functions and developmental processes, and altering uORFs by genome editing provides an easier way to upregulate gene expression than altering coding sequences and promoters [23].

Strawberries are important fruits with a high content of essential nutrients [24]. Though flavor is generally ignored in traditional breeding practices, there is a growing recognition that taste is an important determinant of fruit marketability [25]. However, breeding for improved “taste” is problematic since consumer preference varies along regional, cultural, and age lines. We hypothesized that this problem could be met by creating a series of mutants with a continuum of phenotypic “taste” changes. Taste results from complex interactions between sugars, acids, phenols, and minerals, to which sugars make the largest contribution [26]. To increase sugar content, previous researches have mainly focused on sugar metabolic genes [27], but in general, transcription factors are more likely to exhibit dosage sensitivities than genes encoding metabolic enzymes [28].

Basic (region) leucine zipper proteins (bZIPs) are evolutionarily conserved eukaryotic enhancer-type transcription factors [29, 30]. In *Arabidopsis*, S1-group bZIP genes form heterodimers with C-group members to form signaling hubs [31]. These facilitate metabolic reprogramming upon energy starvation, permitting metabolic adaptation and survival in response to stress [32]. *Arabidopsis* encodes five S1-group bZIP genes (*AtbZIP1*, *AtbZIP2*, *AtbZIP11*, *AtbZIP44*, and *AtbZIP53*), each of which has a highly conserved uORF in the 5′ UTR of its mRNA [22]. The translation of all group S1 members is repressed by sucrose, leading to what is known as sucrose-induced repression of translation (SIRT), and the conserved upstream peptide sequence is essential for this repression [33]. Moreover, these peptides are conserved in many other dicotyledonous and monocotyledonous plants, and a functional SIRT mechanism has even been demonstrated in gymnosperms [22, 34].

In this study, we edited the conserved uORF of the strawberry transcription factor gene *FvebZIPs1.1* and generated seven novel alleles of diverse phenotypic strengths. From these, we produced a series of transgene-free mutants with 35 novel genotypes leading to diverse strawberry sugar contents. Furthermore, the novel genotypes and phenotypes could be maintained in subsequent generations by asexual reproduction. This approach thus provides a way of not only increasing variation but also fine-tuning quantitative traits in asexually reproducing crops.

## Results

### Engineering the conserved uORF of *FvebZIPs1.1* with A3A-PBE

**Fig. 1:**
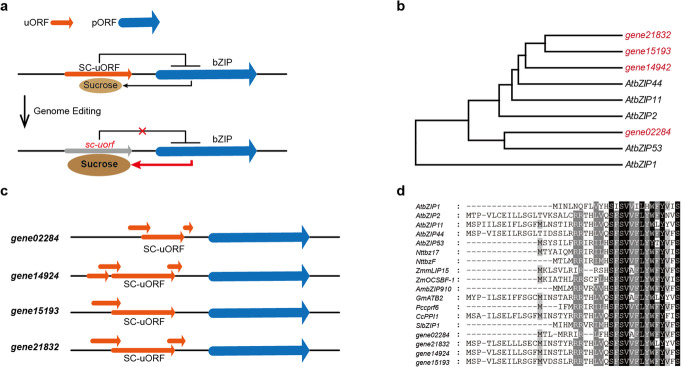
Conserved uORFs of strawberry bZIP genes. **a** Diagram depicting the sucrose-dependent post-transcriptional control of bZIP genes and the strategy for increasing sugar content by engineering the conserved sucrose control uORF (SC-uORF). The mutant sc-*uorf* reduced inhibition of translation of the bZIP gene, leading to increased sugar accumulation. uORF, upstream open reading frame. pORF, primary open reading frame. **b** Phylogenetic tree of the S1 group bZIP genes in *Arabidopsis* and strawberry. The strawberry S1 group bZIP homologs are shown in red. **c** Schematic illustration of the organization of the uORFs in the mRNA of the four strawberry bZIP genes. The uORFs are shown by the yellow lines with arrowheads upstream of the blue line with arrowhead representing the pORF. The longest uORF of each gene is the SC-uORF. **d** Alignment of the conserved SC-uORF amino acid sequences in *Arabidopsis*, strawberry, and other dicotyledonous and monocotyledonous plants. Black box with white letter, 100% identity; dark gray box with white letter, 80% identity; gray box with black letter, 60% identity

Diploid strawberry (*Fragaria vesca*) has been cultivated for centuries in European gardens [35]. We selected diploid strawberry accession Hawaii 4 for trait improvement because of its potential for domestication, asexual reproduction, and the availability of published genomic information [36]. *Arabidopsis* S1-group bZIP genes are reported to fine-tune carbon and nitrogen metabolism to adapt to the prevailing energy supply, and they share a conserved uORF system enabling sucrose-dependent post-transcriptional control [22, 31, 33]. We hypothesized that modulation of the translation of bZIPs by editing the conserved sucrose control uORF (SC-uORF) could be exploited to fine-tune sugar content (Fig. 1a).

First, we performed a BLAST search against the diploid strawberry genome using the *Arabidopsis* S1-group bZIP genes as query. We identified four strawberry bZIP genes closely related to *Arabidopsis* S1-group bZIP genes with putative SC-uORFs in the 5′ leading regions of their mRNAs: *gene02284*, *gene14924*, *gene15193*, and *gene21832* (Fig. 1b). Each of these strawberry genes has more than one uORFs in the 5′ leading region of its mRNA, the longest being the putative SC-uORF (Fig. 1c). Remarkably, phylogenetic analysis of the putative SC-uORF peptide sequence revealed that it was significantly conserved in *Arabidopsis* and in many other dicotyledonous and monocotyledonous plants, for example: tobacco (*Nttbz17*, *NttbzF*), maize (*ZmmLIP15*, *ZmOCSBF-1*), snapdragon (*AmbZIP910*), soybean (*GmATB2*), parsley (*Pccprf6*), pepper (*CcPPI1*), and tomato (*SlbZIP1*) (Fig. 1d). Overexpression of *Nttbz17* and *SlbZIP1* has been shown to increase sugar content in tobacco and tomato [37, 38]. Since *gene02284* is closely related to *Nttbz17* and *SlbZIP1* (Fig. 1d; Additional file 1: Fig. S1) and the regulation mechanism has been elucidated previously [39], we focused on this gene (denoted *FvebZIPs1.1*) in the following studies (Additional file 2: Sequences S1 and Sequences S2).

**Fig. 2:**
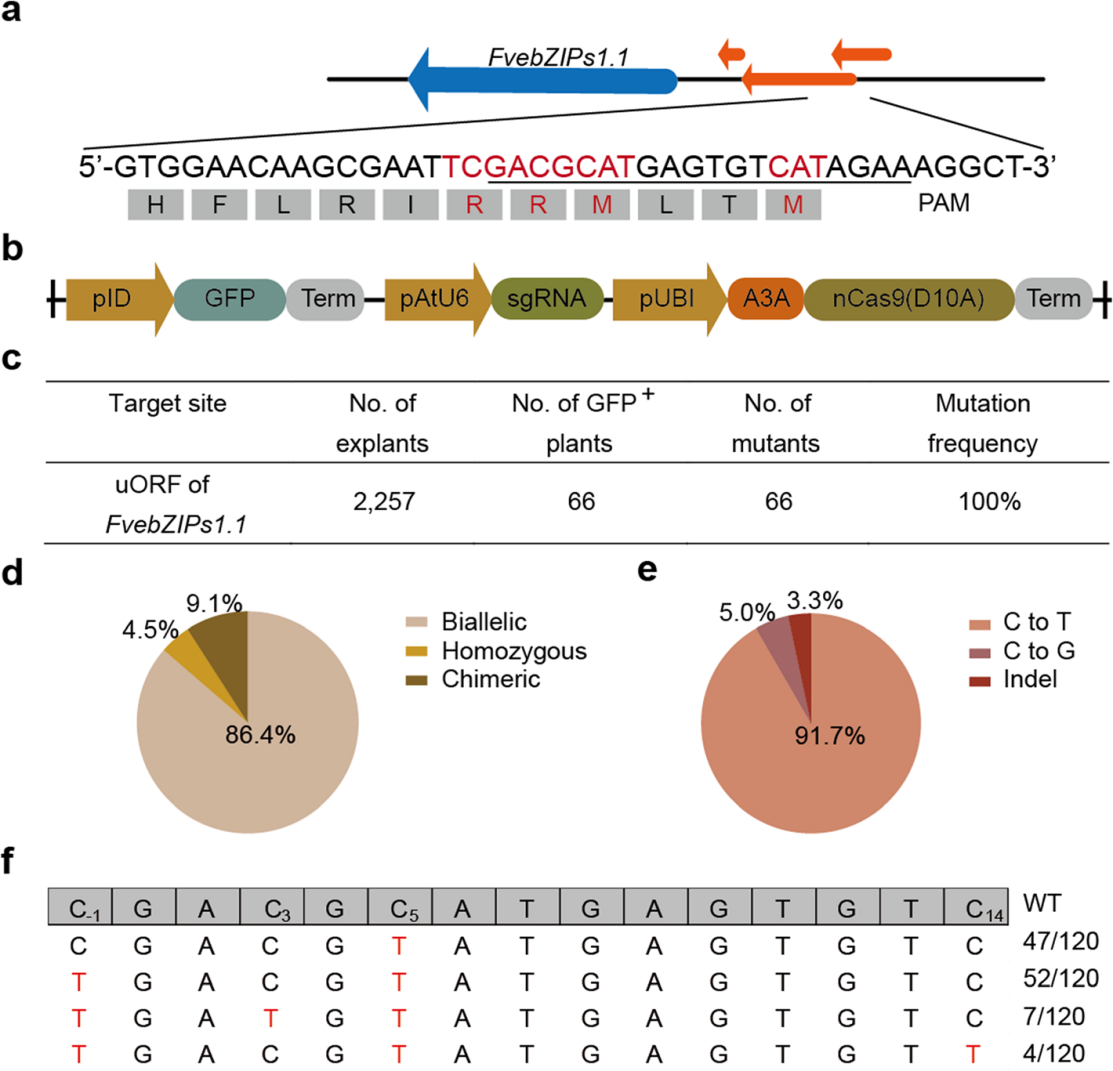
Engineering the SC-uORF of *FvebZIPs1.1* using A3A-PBE. **a** Diagram of the uORFs in *FvebZIPs1.1* and the target of A3A-PBE. Part of the amino acid sequence of the SC-uORF and the corresponding codons in the DNA sequence are shown, and the targeted sequence is underlined. The two ATGs and two codons that encode conserved amino acids (RR) of SC-uORF are marked in red. **b** Schematic of the A3A-PBE vector. GFP fluorescence was used to identify transgenic plants. pUbi, maize *Ubiquitin-1* (Ubi-1) gene promoter. pID, root loci promoter. **c** Frequencies of mutations induced by A3A-PBE in T0 strawberry plants. GFP^+^, GFP fluorescence positive. **d** Proportions of the different types of mutation in T0 strawberry plants. **e** Proportions of C-to-T changes, C-to-G changes, and indels induced by A3A-PBE at the target site. **f** The four different alleles (single and multiple C-to-T conversions) of the uORF among the T0 mutant plants, and their frequencies. Nucleotide substitutions are indicated in red. The letter subscripts on the cytosines in the WT sequence indicate the positions of these bases in the protospacer, counting from the distal end to the protospacer-adjacent motif. Frequency: number of a given allele /total numbers of alleles

We previously developed a highly efficient plant C-to-T base editor which uses the human APOBEC3A deaminase, converts C-to-T efficiently in wheat, rice, and potato, and has a 17-nucleotide editing window [14]. The wide deamination window and efficiency of A3A-PBE suggested that multiple cytidines in a target could be mutated simultaneously and might produce different genetic modifications. We therefore used A3A-PBE to target the conserved SC-uORF of *FvebZIPs1.1* in diploid strawberry. The target region has two start codons (AUG) and two codons that encode a conserved pair of residues of the basic amino acid arginine (Fig. 2a). We inserted a green fluorescent protein (GFP) expression cassette in the A3A-PBE vector (Fig. 2b); after *Agrobacterium*-mediated transformation, we took advantage of GFP fluorescence to identify transgenic plants (Additional file 1: Fig. S2), and obtained 66 first-generation transgenic plants (T0). Sequencing revealed that all 66 plants harbored mutations in the target region (Fig. 2c; Additional file 1: Table S1). Homozygous and biallelic mutants accounted for 90.9% (60/66) of the genetic changes observed (Fig. 2d). No heterozygous mutants were obtained and only 9.1% (6/66) of the mutants were chimeric (Fig. 2d). A total of 120 individual mutant alleles were generated from the homozygous and biallelic mutants, in addition to six chimeric mutants. 91.7% (110/120) of the alleles contained C-to-T mutations only (Fig. 2e), and four combinations of C-to-T substitutions at positions C_− 1_, C_3_, C_5_, and C_14_ of the protospacer were identified in the deamination window spanning positions − 1 to 14 of the protospacer, counting from the distal end to the protospacer-adjacent motif (PAM) (Fig. 2f). The mutation frequencies of individual Cs ranged from 3.3% (C_14_, 4/120) to 91.7% (C_5_, 110/120) (Fig. 2f). To our knowledge, this is the first report of base editing in strawberry.

### The novel alleles cause various levels of translation repression

**Fig. 3:**
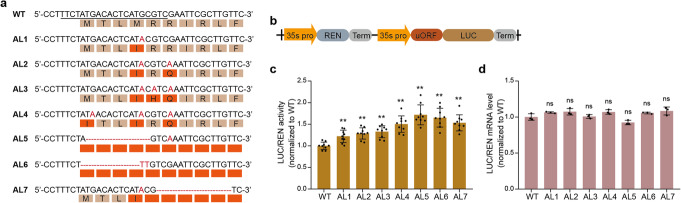
Effect of the novel alleles on translation of the downstream primary open reading frame. **a** The novel alleles generated in the T0 generation. Nucleotide substitutions and small deletions are indicated in red. The amino acid residues of SC-uORF corresponding to the codons in the DNA sequence are indicated by yellow filled boxes. Changed amino acids are indicated by red filled boxes. **b** Schematic of the dual-luciferase reporter vector. 35s pro, cauliflower mosaic virus 35s promoter. REN, *Renilla reniformis* luciferase. LUC, luciferase. **c** Effect of the novel alleles on translation of the pORF in the dual-luciferase reporter system. **d** Effect of the novel alleles on transcription of the pORF in the dual-luciferase reporter system. In **c** and **d**, mean values (±SD) are compared to those for wild-type plants using Student’s *t* tests, **P < 0.01. AL#, allele #

Among the 66 mutants, we identified four novel alleles (AL1-AL4) with multiple combinations of C-to-T substitutions (Fig. 3a; Additional file 1: Fig. S3). By chance, a few alleles harbored small deletions or C-to-G substitutions (Table S1), and we mainly focused on alleles with C-to-T substitutions and small deletions (AL1–AL7) (Fig. 3a; Additional file 1: Fig. S3). The only change in AL1 was a change of the internal start codon (ATG) to ATA, which should reduce translation initiation from that point. In AL2 and AL3, one and two additional arginine codons, respectively, were mutated in addition to the internal ATG. The mutations in these arginine codons changed these strongly basic amino acids to the weakly basic amino acid histidine and the acidic amino acid glutamine, respectively, which should reduce the high isoelectric point of the SC-uORF peptide. In AL4, both the first ATG and internal ATG were mutated to ATA, which would be expected to significantly reduce translation of the SC-uORF. In AL5 and AL6, small deletions removed the two ATGs and should block translation initiation of SC-uORF. In AL7, a small deletion after the internal ATG causes a frame shift and the internal ATG is mutated to ATA. In this case, translation can initiate from the first ATG, but the frame shift disrupts the conserved C terminus. We hypothesized that the different alleles might have different phenotypic strengths.

Next, we tested whether the novel alleles increased translation of the downstream primary open reading frame (pORF), using a dual-luciferase reporter system [40]. The wild-type 5′ leader and seven mutant 5′ leaders described above were each cloned upstream of the luciferase (LUC) coding region in an expression cassette driven by the 35S promoter (Fig. 3b). The resulting constructs also harbored a second 35S-promoter-driven cassette expressing *Renilla reniformis* luciferase (REN) as an internal vector control. The eight constructs were transiently expressed in strawberry fruits by *Agrobacterium*-mediated transformation, and we calculated LUC/REN activities and LUC/REN mRNA levels. The mutant leaders with small deletions that remove the two AUG codons (AL5 and AL6) consistently generated high LUC/REN activity levels, 71.7% and 65.1% higher than the WT 5′ leader (Fig. 3c). The 5′ leaders with the two ATGs mutated to ATA (AL4) were also effective and increased WT LUC/REN activity by 50.2%. The small deletions after the internal ATG causing a frame shift (AL7) had a similar effect as AL4, yielding 53.3% higher activity than the WT 5′ leader. The LUC/REN activities of AL1, AL2, and AL3 were 22.0%, 28.7%, and 32.8% higher, respectively, than that of the WT, while quantitative RT-PCR assays revealed that the LUC/REN mRNA ratios of transcripts from the various constructs did not differ significantly (Fig. 3d). These data indicate that mutations in both the start codon and conserved C terminus of SC-uORF are generally effective in upregulating pORF translation.

### Fine-tuning sugar content by combining diverse alleles

**Fig. 4:**
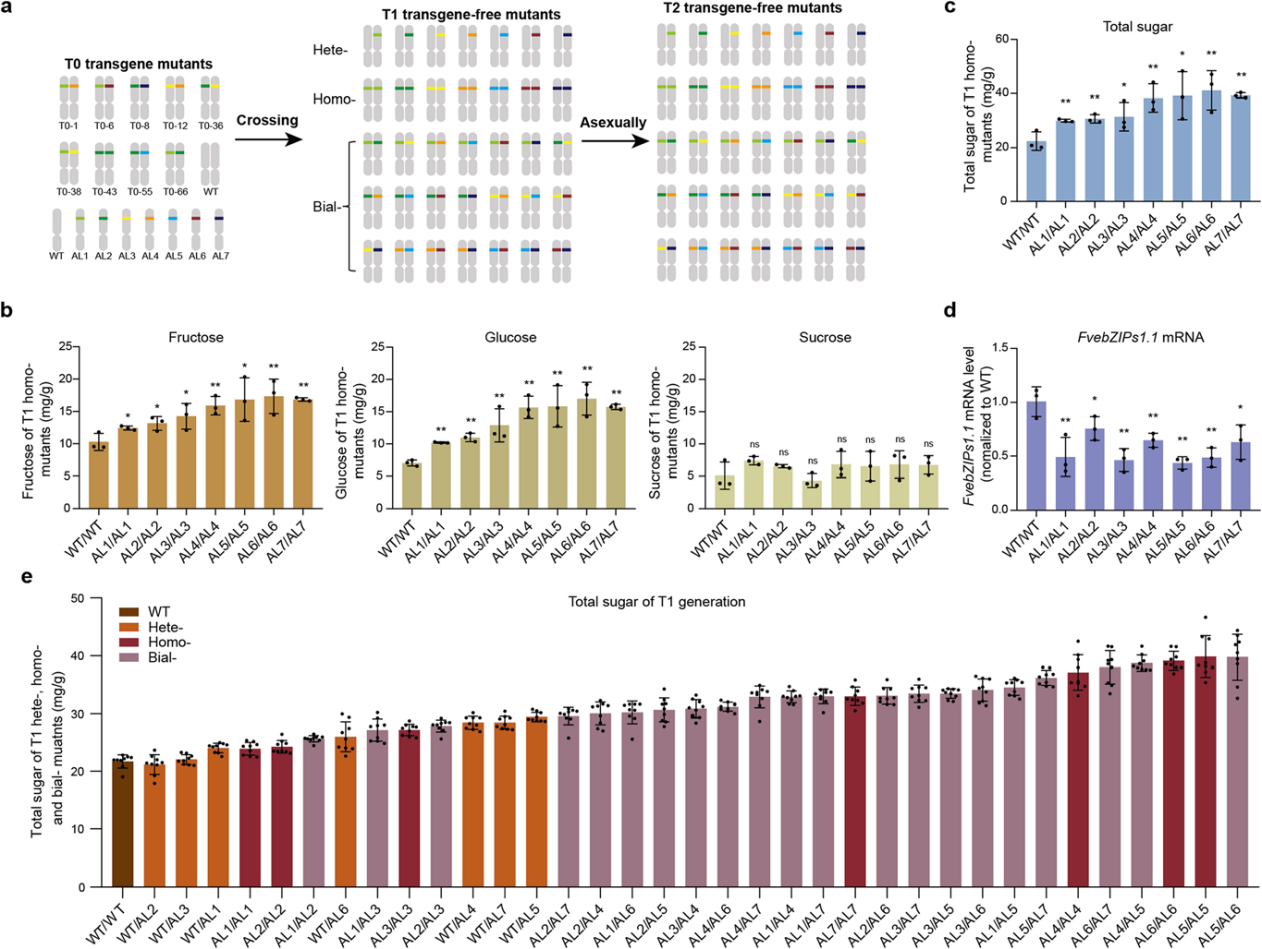
Sugar content of the novel genotypes. **a** Model showing the strategy for enriching genotype diversity, and the advantage of asexual reproduction for fixing novel genotypes. **b** Fructose, glucose, and sucrose contents of the seven T1 homozygous mutants and WT. **c** Total sugar contents of the seven T1 homozygous mutants and WT. **d** Transcription of *FvebZIPs1.1* in the seven homozygous strains and WT. **e** Total sugar contents of the 35 novel genotypes  and WT in the T1 generation (means ± SD). In **b** to **d**, mean values (±SD) are compared to those for wild-type plants using Student’s *t* tests, **P < 0.01, *P < 0.05. AL#/AL#, combination of allele# and allele#. Hete-, heterozygote. Homo-, homozygote. Bial-, biallelic

To confirm the phenotypic effects of these alleles *in planta*, and test whether their phenotypic effects might be additive in heterozygous and biallelic plants, we generated more than 4000 T1 seedlings by crossing the biallelic and homozygous T0 mutants to each other and to WT. Transgene-free seeds were detected by GFP fluorescence, and GFP fluorescence-negative seeds (more than 900) were planted individually. We also tested for the presence of transgene fragments in the GFP fluorescence-negative seedlings by PCR assays and the PCR assays generally confirmed the GFP fluorescence assays (Additional file 1: Fig. S4). Transgene-free seedlings identified by both the GFP fluorescence and PCR assays were genotyped by sequencing, and we obtained a series of mutants with 35 novel genotypes that covering the complete set of combinations of all the alleles (Fig. 4a; Additional file 1: Fig. S5). All the genotypes generated in T1 could be fixed in T2 generation, as strawberry is a typical asexually reproducing crop. We evaluated the potential for off-target effects in T1 mutants homozygous for the seven alleles. We searched the genomic sequence for all target sites that contained sequences with up to a 4-nt mismatch and sequenced the top three potential off-target sites in the homozygous mutants. We found no off-target mutations in any of the homozygous mutants (Additional file 1: Table S2 and S3).

We cultivated these T1 mutants in a self-contained, climate-controlled room and harvested mature fruits for sugar content tests 20 days after pollination. We first examined the sugar contents of T1 homozygous mutants. Quantitative measurements showed that the fructose contents of homozygous mutants for AL1–AL7 were 20.9%, 28.0%, 38.6%, 54.6%, 63.8%, 68.7%, and 63.9%, respectively, higher than that of WT plants, respectively, and, for glucose, they were 44.8%, 56.2%, 82.7%, 122.4%, 124.6%, 141.5%, and 123.5% higher (Fig. 4b). In contrast, their sucrose contents did not differ significantly (Fig. 4b). Total sugar contents were 33.9%, 36.9%, 40.3%, 71.1%, 74.9%, 83.6%, and 75.3% higher, respectively, than in WT plants (Fig. 4c). The significantly higher fructose and glucose, but not sucrose contents in the mutants may be due to the high and constant invertase activity in fruits. The major function of the high and constant invertase activity in fruits is to maintain high cellular hexose concentrations. The hydrolysis of sucrose in the vacuole and in the intercellular space allows more efficient storage of sugar in these compartments [41]. Thus, engineering the conserved SC-uORF of *FvebZIPs1.1* is an efficient way to increase sugar content in strawberry. Although previous studies have shown that uORFs affect translational regulation of downstream main open reading frames without affecting transcription [16, 17, 22], our quantitative RT-PCR assays revealed that the levels of *FvebZIPs1.1* transcripts in the seven homozygous mutants were lower than in the WT (Fig. 4d). It is possible that the increased sugar content of the mutants leads to repression of *FvebZIPs1.1* mRNA transcription, as previously reported in *Arabidopsis* [32, 42]. Organic acids (such as citric acid and malic acid) are key components impacting fruit flavor. The analyses showed that the citric acid was slightly lower in homozygous mutants for AL5 and AL6 than that in WT, but malic acid contents in the seven homozygous mutants were not affected (Additional file 1: Fig. S6).

To see whether the phenotypic effects of the mutant alleles might combine to fine-tune sugar content, we evaluated the total sugar contents of the fruits of heterozygous, homozygous, and biallelic T1 plants. Nine mature fruits of each genotype were tested. The different combinations of these alleles were indeed found to generate a continuum of sugar contents (Fig. 4e). The sugar contents of biallelic combinations of AL1, AL2, and AL3 were 18% (AL1/AL2) to 28% (AL2/AL3) higher than that of WT plants. The sugar contents of biallelic combinations of AL4, AL5, AL6, or AL7 were more strongly increased; they were 43.7% (AL4/AL6) to 83.1% (AL5/AL6) higher than that of WT plants. Notably, the sugar contents of biallelic plant combinations involving a weak allele, such as AL1, AL2, AL3, and a strong allele, such as AL4, AL5, AL6, and AL7, were 36.2% (AL2/AL7) to 59.0% (AL1/AL5) higher than that of WT plants. Sugar contents were particularly high in the combinations AL6/AL7, AL4/AL5, AL6/AL6, AL5/AL5, and AL5/AL6 (75.0%, 78.3%, 80.2%, 83.6%, and 83.1% higher, respectively, than that of WT plants). These results show that combinations of the mutant alleles can fine-tune sugar contents in strawberry.

Strawberry is a typical asexually reproducing crop that produces new plants from modified stems called stolons. All the genotypes generated in T1 could be immediately fixed by asexual reproduction. To determine whether the different sugar contents of the T1 plants were transmitted to subsequent generations by asexual reproduction, we generated a T2 generation of the novel genotypic combinations from strawberry stolons of the transgene-free T1 mutants and cultivated them by the same farming method as the T1 generation. We observed a similar gradual increase in terms of sugar content as in the T1 generation (Additional file 1: Fig. S7), confirming that the phenotypic diversity generated in the T1 can be transmitted to the T2 by asexual reproduction.

**Fig. 5:**
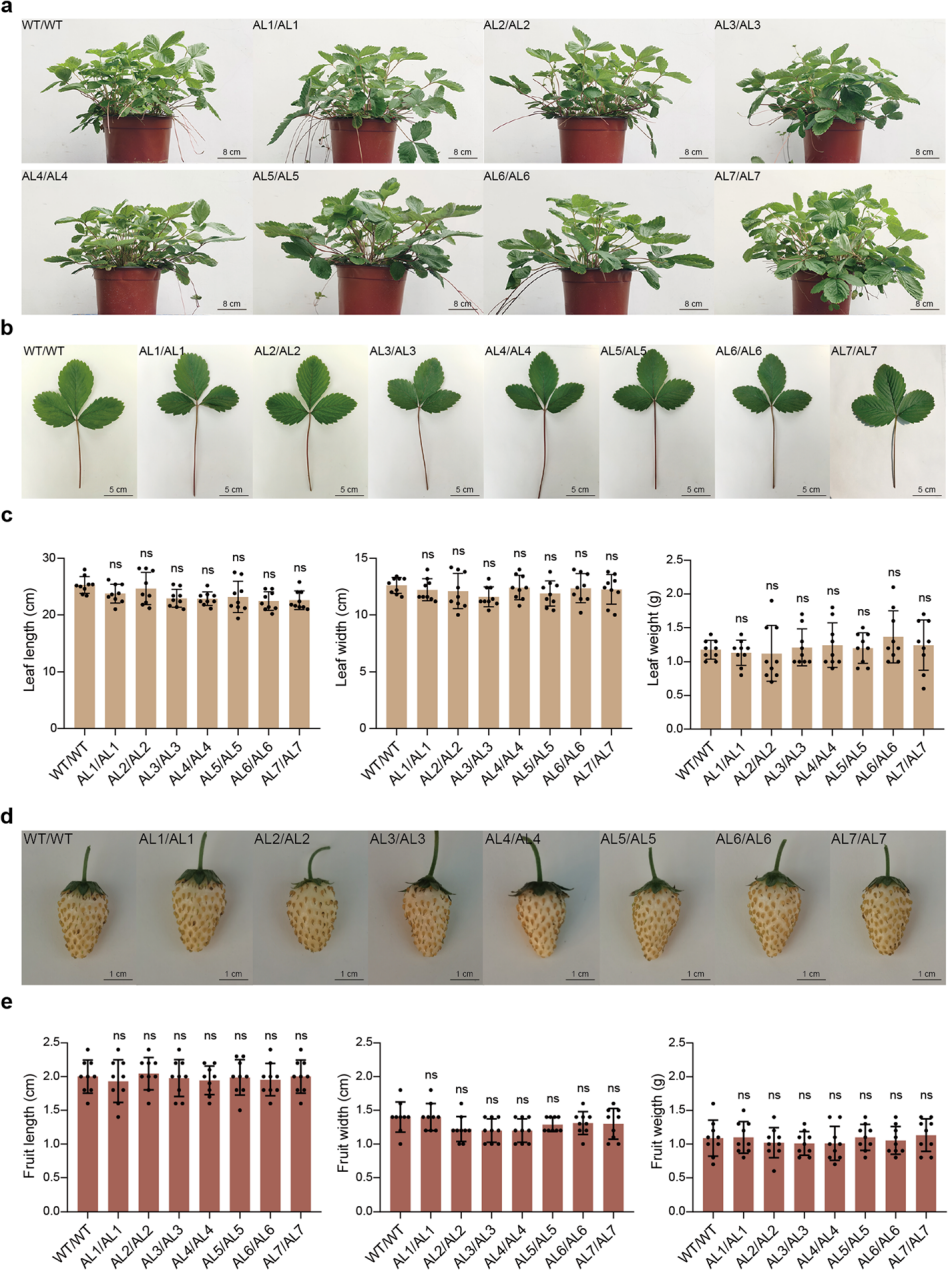
Growth parameters of homozygotes. **a** Appearance of the seven homozygous mutant plants and WT. **b** Leaves of the seven homozygous genotypes and WT. **c** Leaf lengths, widths, and weights. **d** Fruits of the eight strains. **e** Fruit lengths, widths, and weights. In **c** and **e**, mean values (±SD) are compared to those of wild-type plants using Student’s *t* tests. AL#/AL#, homozygote for allele #

We have thus shown that genomic editing of the SC-uORF of *FvebZIPs1.1* can increase the sugar content of strawberry fruit. However, sugars have been shown to have multiple roles in plant development [43]. To evaluate the potential application of the *FvebZIPs1.1* uORF mutants generated by our fine-tuning strategy, we examined their agricultural traits. As shown in Fig. 5a–c, the appearance of the *FvebZIPs1.1* uORF mutants was healthy, their leaf shapes and leaf areas, which are determined by the length and width of the mature leaves, were the same as in the wild-type, and no dwarfed plants, slow growth rates, and failed pollination were observed. Fruit yield which is the key factor determining the economic value of the strawberry is closely related to fruit size and fruit weight. Therefore, we examined the fruit sizes and fruit weights and found that the mutant fruits were well-developed, and fruit sizes and fruit weights were similar to the wild-type (Fig. 5d, e). Together, these results indicate that the increased sugar content of the *FvebZIPs1.1* uORF mutants is not accompanied by negative traits that reduce the economic value of strawberry, suggesting that our strategy can be used to improve the sugar quality of fruits.

## Discussion

Fine-tuning of quantitative traits is highly valued by breeders as it provides a way to harness vital characteristics for breeding without causing severe growth impairment [1, 6]. Diverse mutants with fine-tuned quantitative traits can also greatly extend trait variation. The natural occurrence of weak alleles affecting vital traits has produced major advances in evolution, domestication, and breeding [44, 45]. However, the limited number of available alleles makes it difficult to fine-tune quantitative traits. In this study, we showed that strawberry sugar content could be fine-tuned by creating combinations of diverse uORF alleles of *FvebZIPs1.1*. Significantly, the novel genotypes and phenotypes were maintained in subsequent generations through asexual reproduction. This study shows that the creation of a continuum of values of a quantitative trait by genome editing technology in asexually reproducing crops offers tremendous opportunities for trait improvement.

Sustaining energy homeostasis is crucial to every living being. The C/S1 bZIP network is thought to act as a regulatory hub orchestrating plant energy homeostasis [31]. Ectopic expression of *Arabidopsis* S1-group bZIPs (*AtbZIP2*, *AtbZIP11*, *AtbZIP44*, and *AtbZIP53*) leads to severe impairment of growth [31]. It has also been reported that constitutive transgenic expression of *Nttbz17* lacking its 5′-leader region leads tobacco plants with thicker leaves [37]. In this study, we engineered the SC-uORF of *FvebZIPs1.1* by using a base editor. We fine-tuned sugar contents in strawberry fruits without severely impairing plant growth.

We have thus demonstrated a way of optimizing the sugar content of strawberries by modifying the translation of a bZIP gene. Our results suggest a general model for increasing the sugar content of plants based on the strong conservation of the SC-uORF. The importance of bZIP transcription factors is highlighted by their evolutionary conservation, as bZIPs can be traced back to algal ancestors, and a functional SIRT system has been demonstrated in gymnosperms [34]. One potential application of our strategy might be in sugarcane. Sugarcane is the most important sugar-producing crop, and it is also propagated asexually [46].

There are thus two main reasons for believing in the widespread possibility of fine-tuning vital traits and enriching genetic variation by creating and combining diverse alleles in asexually reproducing crops. First, thanks to the great advances in gene editing using the CRISPR system, the opportunity now exists to rapidly create targeted mutations to fine-tune and optimize yield in many crops [47–50]. Second, the available evidence suggests that dosage effects may exist in many important conserved pathways [1, 4, 6, 7]. Our work highlights the advantage of asexually reproducing crops for fine-tuning quantitative traits.

Although we found no mutations in potential off-target sites, sgRNA-independent off-target mutations may exist [51]. Very recently, the two newly developed cytosine base editors, A3Bctd-VHM-BE3 and A3Bctd-KKR-BE3, exhibited high specificity and precision in plants. sgRNA-independent DNA off-target edits can be dramatically reduced by using them [52].

## Conclusions

Engineering the conserved upstream open reading frame of the gene *FvebZIPs1.1* by genome editing increased the sugar content of strawberry. Sugar content was fine-tuned by combining diverse alleles to generate a series of novel mutants with diverse phenotypic strengths. All the novel genotypes and phenotypes were maintained in subsequent generations by asexual reproduction. Using strawberry as an example, we have shown that combining diverse alleles created by genome editing offers tremendous opportunities for trait improvement in asexually reproducing crops.

## Methods

### Construction of the A3A-PBE and sgRNA expression vector for strawberry transformation

The GFP expression cassette of the Gateway vector pH7WG2D [53] was cloned and fused to the SpeI-digested vector A3A-PBE [14] with the primer set ProGFP-F and ProGFP-R (Additional file 1: Table S4) using the Gibson cloning method [54]. The gRNA target site was selected manually, based on the sequence of SC-uORF. To check for specificity, BLAST analyses of the gRNA target site were performed against the strawberry genome [36]. The sgRNA expression cassette including the uORF-targeting sequence was constructed from pYLsgRNA-AtU6-29 [55] by adaptor ligation and PCR amplification with primers containing the gRNA sequence (Additional file 1: Table S4). The sgRNA expression cassette was then subcloned into BsaI-digested A3A-PBE-GFP vector by standard Golden Gate assembly [56], and the resulting constructs were used to transform diploid strawberry (*Fragaria vesca*, Hawaii4) by *Agrobacterium*-mediated transformation (see below).

### *Agrobacterium*-mediated transformation of strawberry leaf explants

*Agrobacterium tumefaciens* strain EHA105 was transformed with the final binary vectors. Transformation of leaf explants of diploid strawberry was conducted as reported [57]. In brief, leaf explants were incubated with *Agrobacterium* suspension for 20 min at room temperature. The explants were blotted dry, transferred to co-cultivation medium, and incubated at 25 °C in the dark for 3 days. They were then rinsed in washing-off medium and transferred to non-selective shoot induction medium. GFP fluorescence was used to identify transgenic plants.

### Genotyping of the transgenic plants

Leaf samples were collected from GFP fluorescence-positive plants, and genomic DNA was extracted with DNA Quick Plant System (Tiangen Biotech, Beijing, China). The targeted sequences were amplified with specific primers (Additional file 1: Table S4), and Sanger sequencing was performed to identify mutations in the target region. Individual mutants were genotyped by sequencing cloned PCR products. At least four clones per sample were sequenced.

### Dual-luciferase reporter system

The wild-type 5′ leader and seven mutant 5′ leaders were each cloned upstream of the luciferase (LUC) coding region in an expression cassette driven by the 35S promoter in pGreenII0800-LUC vector [40]. The eight constructs were transiently expressed in strawberry fruits by *Agrobacterium*-mediated transformation. In brief, *Agrobacterium tumefaciens* strain EHA105, containing the dual-luciferase reporter vector, was resuspended in infection buffer (10 mM MgCl_2_, 10 mM MES, pH 5.6, and 200 μM acetosyringone). Attached fruits of almost identical sizes and shapes were selected, and the *A. tumefaciens* suspension was evenly injected into the fruits with a syringe until the whole fruit became transparent [58]. The fruits were collected 3 days after transfection. LUC/REN activities and LUC/REN mRNA levels were calculated as described previously [23].

### Recovery of transgene-free progeny from T0 plants

To generate a series of transgene-free mutants of diverse genotypes, the T0 mutants were crossed according to their genotypes. GFP fluorescence-negative seeds were planted into soil and grown under standard greenhouse conditions. Genomic DNA was extracted with a DNA Quick Plant System (Tiangen Biotech, Beijing, China). Transgene fragments in GFP fluorescence-negative seedlings were further detected by PCR assay. The targeted sequences were amplified with specific primers (Additional file 1: Table S4), and sequenced to identify the genotypes of the corresponding plants.

### Measurement of sugar content

We cultivated T1 and T2 mutants in a self-contained, climate-controlled LED farm system. Mature fruits were harvested 20 days after pollination. At least nine fruits of each genotype were tasted. The sugar content of the strawberry fruits was measured using an anthrone reagent kit (Jiancheng Biotech, Nanjing, China) and high-performance liquid chromatography (HPLC).

### Statistical analysis

All numerical values are presented as means ± SD. The significance of differences between the WT control and the relevant mutants was tested by two-tailed Student’s *t* tests.

## Supplementary information

**Additional file 1: Figure S1.** Phylogenetic tree of the *SlbZIP1*, *Nttbz17* and four strawberry bZIP genes. **Figure S2.** Detection of transgenic shoots by GFP fluorescence in the T0 generation. **Figure S3.** Sanger sequencing chromatograms of alleles 1–7. AL1-AL7, allele 1 - allele 7. **Figure S4.** Detection of transgene-free T1 mutants. **Figure S5.** Sanger sequencing chromatograms of representative T1 plants of all genotypes. **Figure S6.** Citric acid and malic acid contents of WT and homozygous mutants. **Figure S7.** Total sugar contents of the 35 novel genotypes  and WT in the T2 generation. **Table S1.** Genotypes of 66 mutant lines in the T0 generation. **Table S2.** Potential off-target sites. **Table S3.** Potential off-target analyze for homozygous mutants. **Table S4.** Sequences of primers used to construct vectors and identify mutation events.

**Additional file 2. **Sequences S1. 5′ untranslated region (UTR) of the gene *FvebZIPs1.1*. Sequences S2. Coding sequences of *FvebZIPs1.1*.

**Additional file 3.** Review history.
