# Correction: Fine-tuning sugar content in strawberry

**DOI:** 10.1186/s13059-025-03894-y

**Published:** 2025-12-24

**Authors:** Sinian Xing, Kunling Chen, Haocheng Zhu, Rui Zhang, Huawei Zhang, Bingbing Li, Caixia Gao

**Affiliations:** 1 State Key Laboratory of Plant Cell and Chromosome Engineering, Center for Genome Editing, Institute of Genetics and Developmental Biology, Innovation Academy for Seed Design, Chinese Academy of Sciences, Beijing, China; 2 College of Advanced Agricultural Sciences, University of Chinese Academy of Sciences, Beijing, China; 3 College of Horticulture, China Agricultural University, Beijing, China

**Correction: Genome Biol 21**,** 230 (2020)**

**https://doi.org/10.1186/s13059-020-02146-5**

Following publication of the original article [1], the authors identified an error in Fig. 1b, “gene14942” should be adjusted to “gene14924”,

The incorrect Fig. 1b is given below.


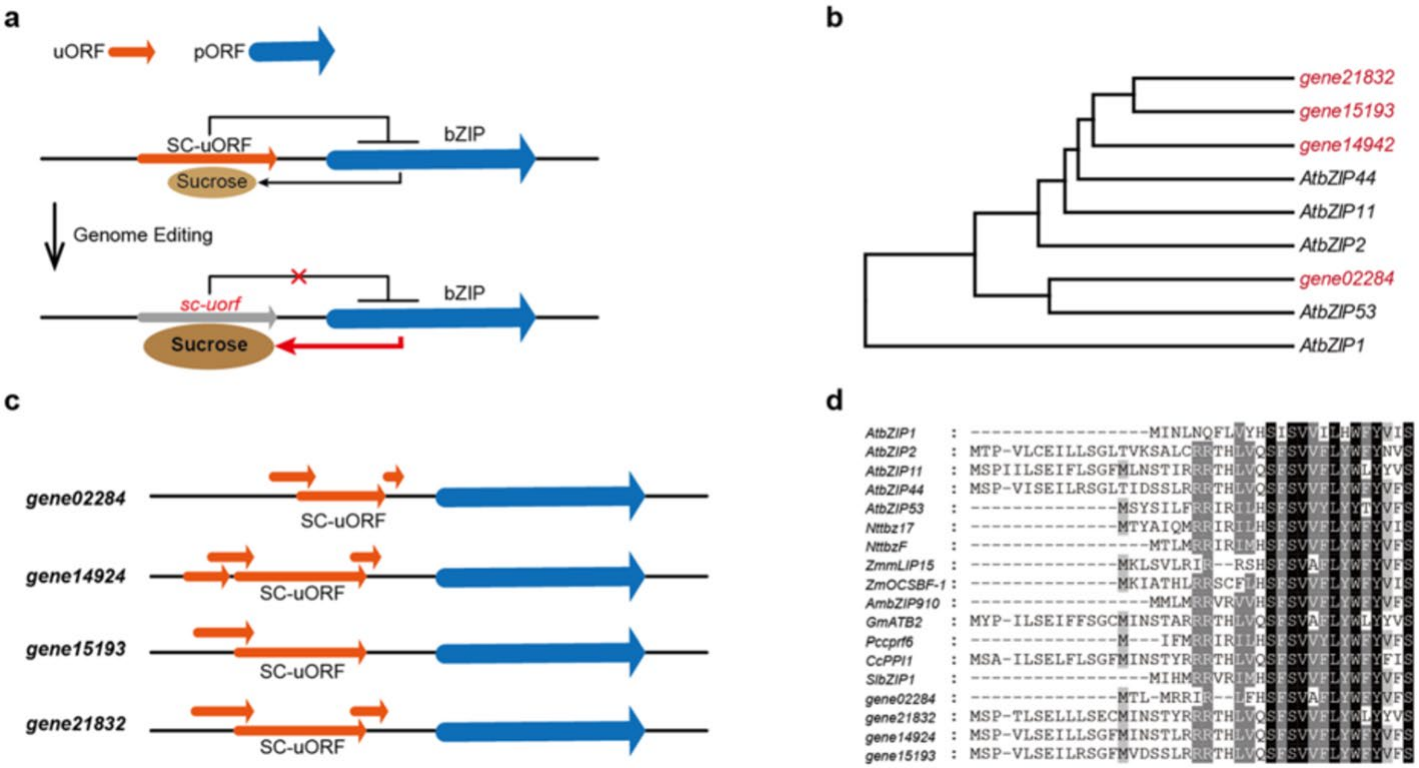


The correct Fig. 1b is given below.


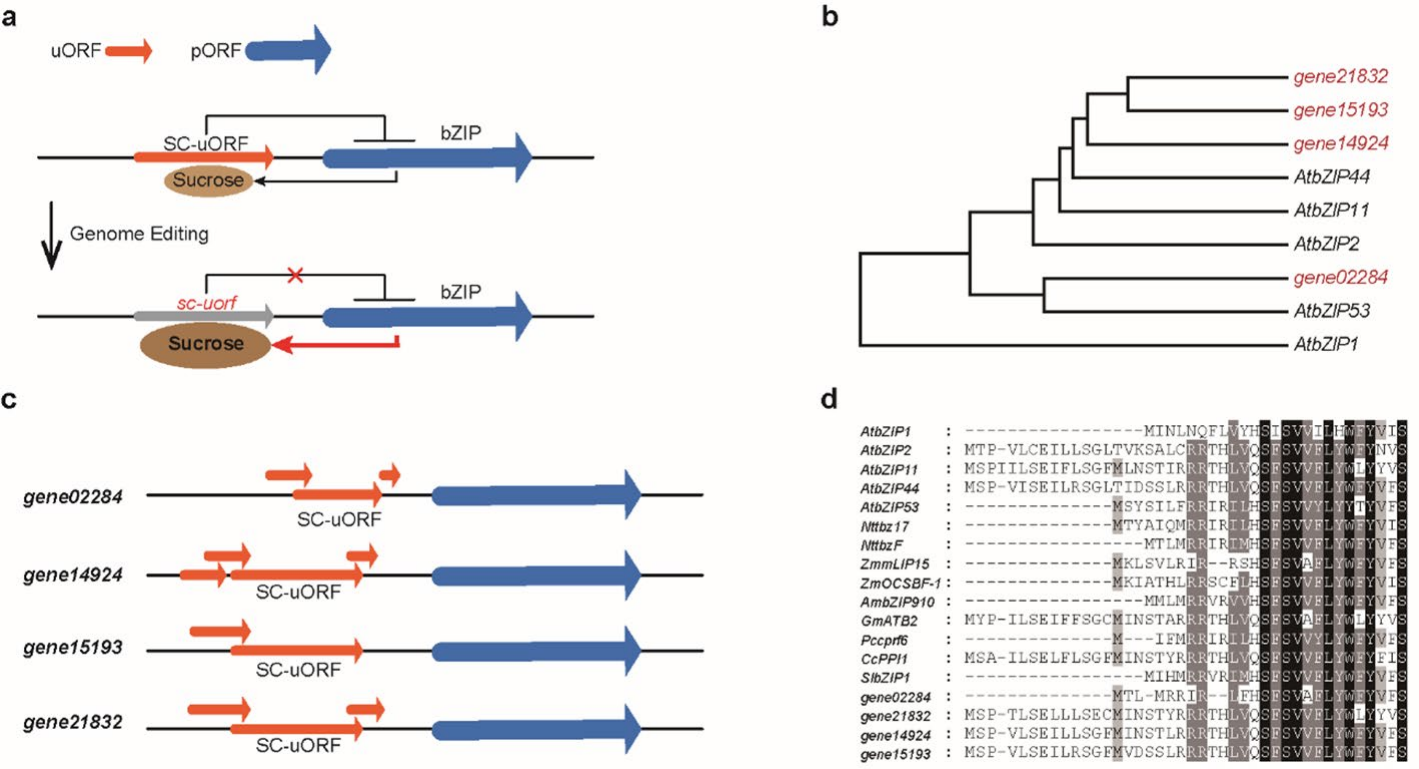


The authors identified an error in Fig. 5d, the pictures of AL1/AL1 and WT/WT were duplicated. We have replaced AL1/AL1 with the correct picture.

The incorrect Fig. 5d is given below.


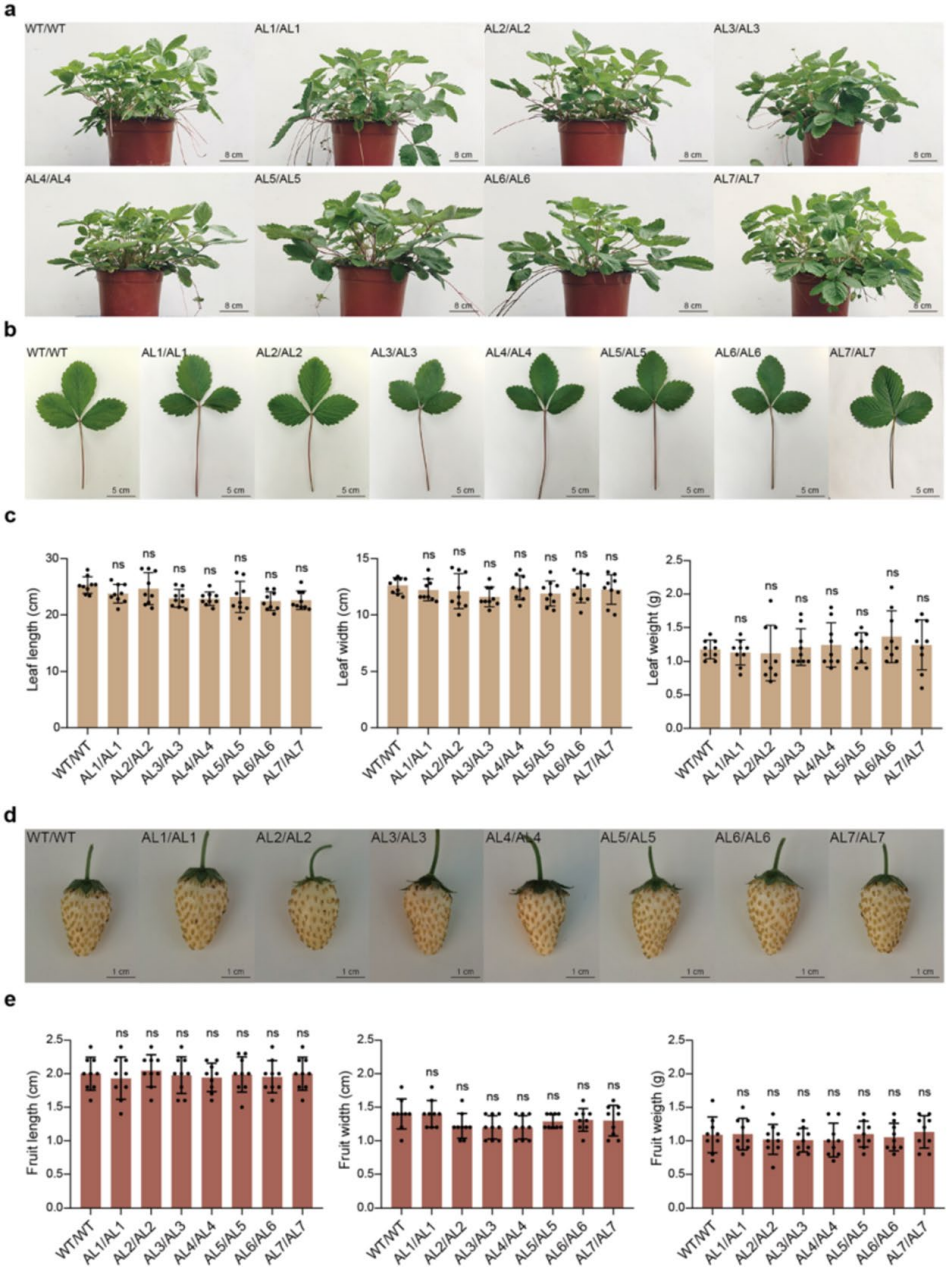


The correct Fig. 5d is given below.


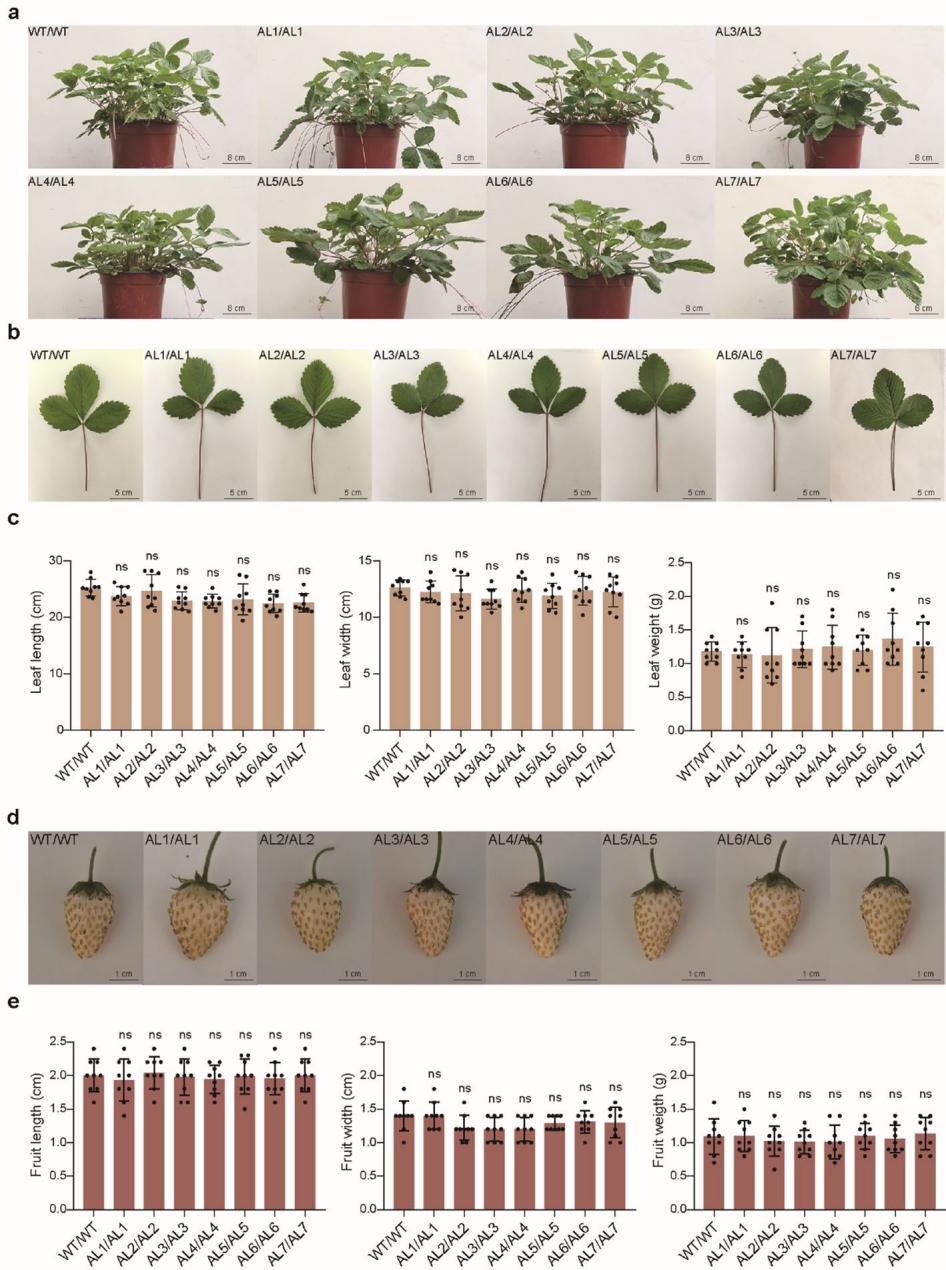


These errors do not affect the main results and conclusions of the paper.
