# Supplementary material for: Fine-tuning sugar content in strawberry
Source: Genome Biol. 2020 Sep 3;21:230. doi: 10.1186/s13059-020-02146-5 (PMC7470447; doi:10.1186/s13059-020-02146-5)
Supplement: Supplementary file 1 — Additional file 1: Figure S1. Phylogenetic tree of the SlbZIP1, Nttbz17 and four strawberry bZIP genes. Figure S2. Detection of transgenic shoots by GFP fluorescence in the T0 generation. Figure S3. Sanger sequencing chromatograms of alleles 1–7. AL1-AL7, allele 1 - allele 7. Figure S4. Detection of transgene-free T1 mutants. Figure S5. Sanger sequencing chromatograms of representative T1 plants of all genotypes. Figure S6. Citric acid and malic acid contents of WT and homozygous mutants. Figure S7. Total sugar contents of the 35 novel genotypes and WT in the T2 generation. Table S1. Genotypes of 66 mutant lines in the T0 generation. Table S2. Potential off-target sites. Table S3. Potential off-target analyze for homozygous mutants. Table S4. Sequences of primers used to construct vectors and identify mutation events. [file 13059_2020_2146_MOESM1_ESM.docx]

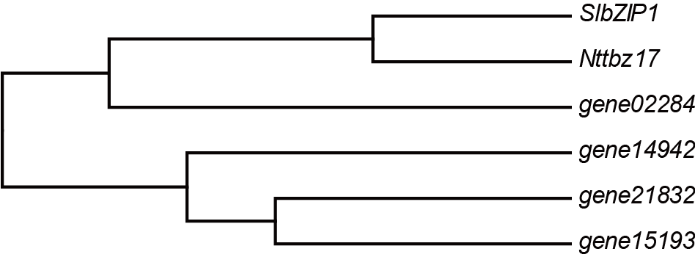


**Figure S1.** Phylogenetic tree of the *SlbZIP1*, *Nttbz17* and four strawberry bZIP genes.


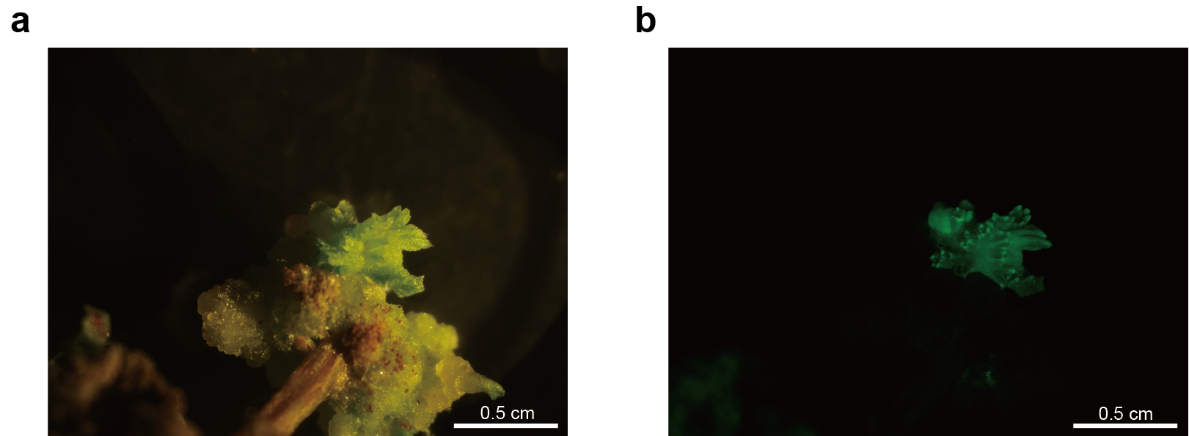


**Figure S2.** Detection of transgenic shoots by GFP fluorescence in the T0 generation. **a.** Bright field. **b.** GFP fluorescence.

**
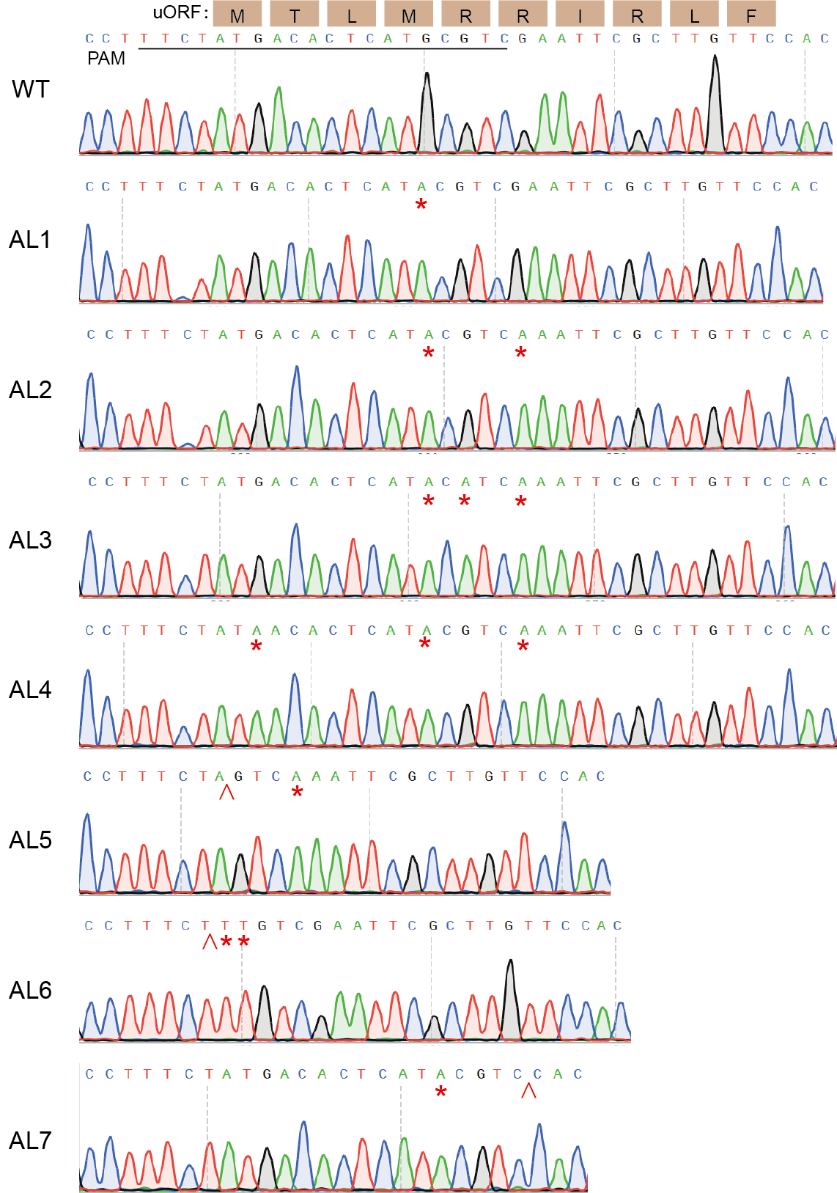
**

**Figure S3.** Sanger sequencing chromatograms of alleles 1 - 7. AL1-AL7 represent mutant allele 1 - allele 7. The Target site is underlined. Altered bases are marked by red asterisks. Deletions are marked by red open arrows.


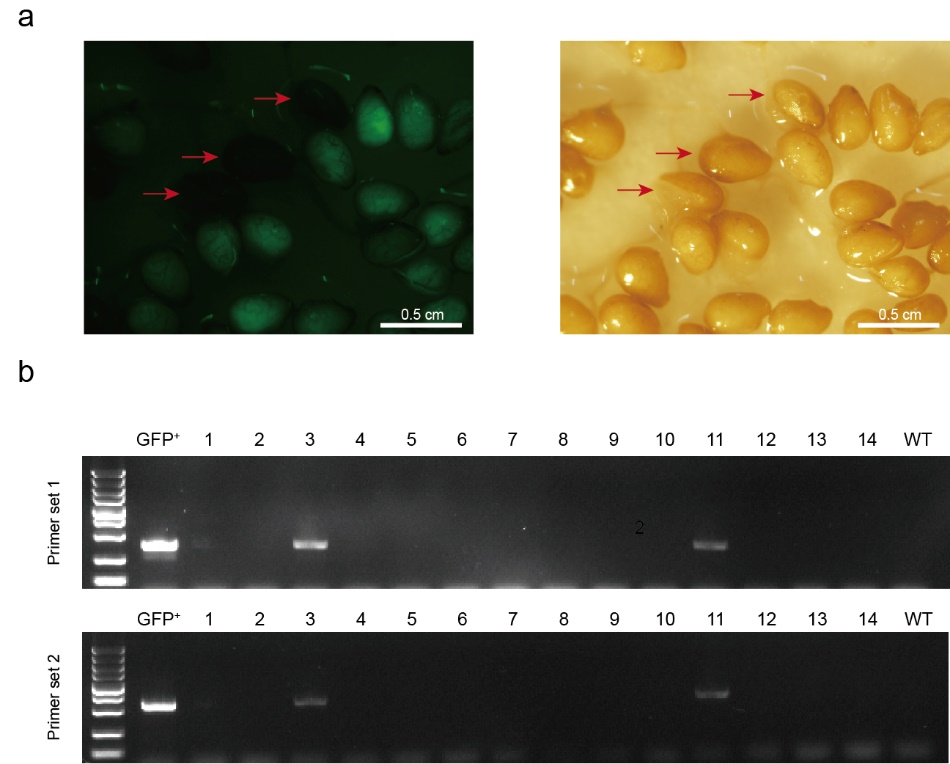


**Figure S4.** Detection of transgene-free T1 mutants. a. Detection of transgene-free seeds by GFP fluorescence. Arrows indicate seeds tested negative for GFP fluorescence. **b.** PCR Detection of transgene-free plants generated from 14 representative seeds that were tested negative for GFP fluorescence. Primer set 1 was used to detect Cas9(D10A) insertion. Primer set2 was used to detect GFP insertion. Lanes labeled with WT or GFP^+^ show the PCR fragments amplified from a WT and GFP fluorescence positive plant, respectively.


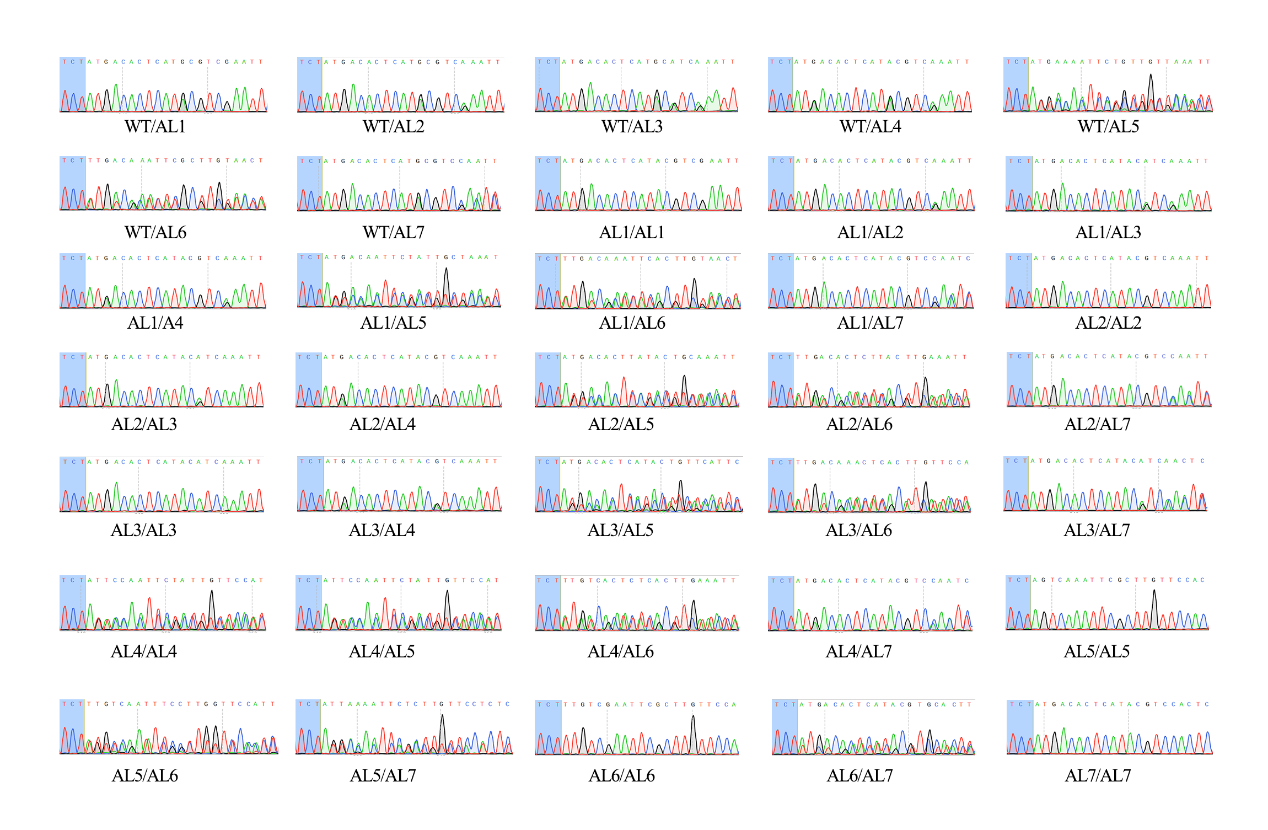


**Figure S5.** Sanger sequencing chromatograms of representative T1 plants of all genotypes.


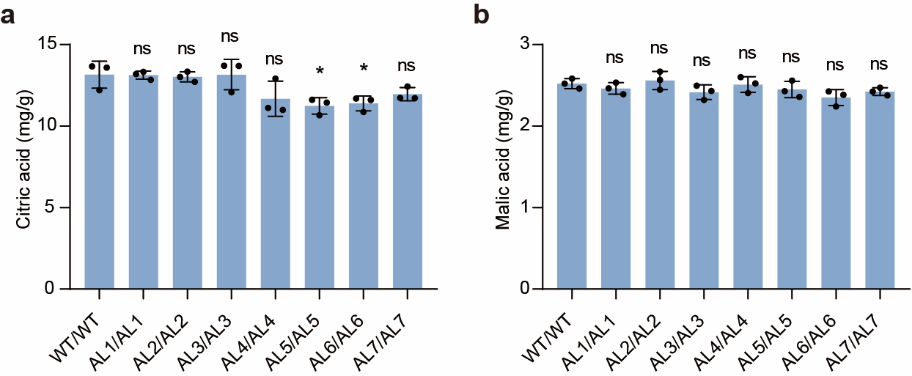


**Figure S6.** Citric acid and malic acid contents of WT and homozygous mutants. Citric acid (**a**) and malic acid (**b**) are measured by HPLC. In **a** and **b**, mean values (± SD) are compared to those for WT plants using Student’s t tests; *, P < 0.05; ns, no significant difference.


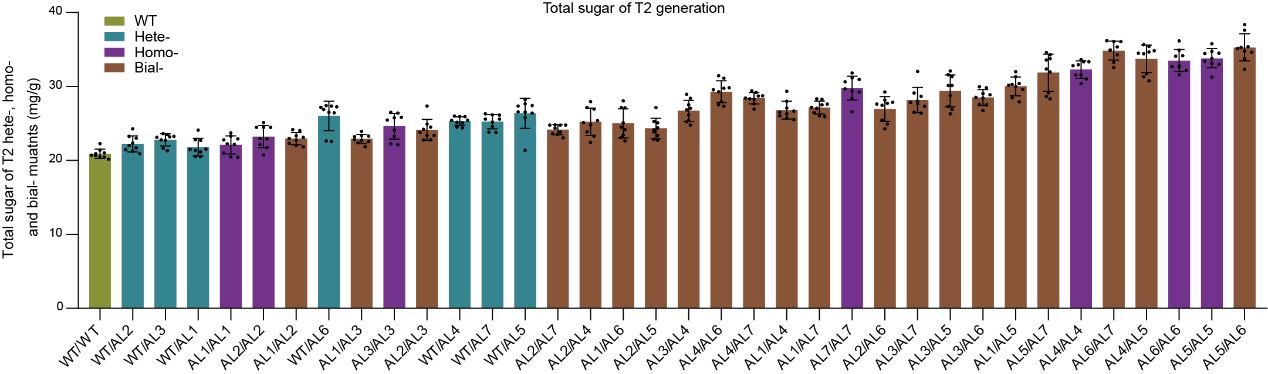


**Figure S7.** Total sugar contents of the 35 novel genotypes and WT in the T2 generation. (means ± SD). AL#/AL#, combination of allele# and allele#. Hete-, heterozygote. Homo-, homozygote. Bial-, biallelic.

**Table S1.** Genotypes of 66 mutant lines in the T0 generation.

| T0 plant ID | Mutant type | Genotype |
| --- | --- | --- |
| WT | ATGACACTCATGCGTCGAATTCGCTTGTTCCAC  ATGACACTCATGCGTCGAATTCGCTTGTTCCAC | WT |
| T0-1 | ATAACACTCATACGTCAAATTCGCTTGTTCCAC  ATGACACTCATACGTCGAATTCGCTTGTTCCAC | Biallelic |
| T0-2 | ATGACACTCATACGTCAAATTCGCTTGTTCCAC  ATGACACTCATACGTCGAATTCGCTTGTTCCAC | Biallelic |
| T0-3 | ATGACACTCATACGTCAAATTCGCTTGTTCCAC  ATGACACTCATACGTCGAATTCGCTTGTTCCAC | Biallelic |
| T0-4 | ATGACACTCATACGTCAAATTCGCTTGTTCCAC  ATGACACTCATACGTCGAATTCGCTTGTTCCAC | Biallelic |
| T0-5 | ATGACACTCATACGTCAAATTCGCTTGTTCCAC  ATGACACTCATACGTCGAATTCGCTTGTTCCAC | Biallelic |
| T0-6 | TT----------------------GTCGAATTCGCTTGTTCCAC  ATGACACTCATACGTCGAATTCGCTTGTTCCAC | Biallelic |
| T0-7 | ATGACACTCATACGTCAAATTCGCTTGTTCCAC  ATGACACTCATACGTCGAATTCGCTTGTTCCAC | Biallelic |
| T0-8 | ATGACACTCATACGT------------------------------CAC  ATGACACTCATACGTCGAATTCGCTTGTTCCAC | Biallelic |
| T0-9 | ATGACACTCATACGTCAAATTCGCTTGTTCCAC  ATGACACTCATACGTCGAATTCGCTTGTTCCAC | Biallelic |
| T0-10 | ATGACACTCATACATCAAATTCGCTTGTTCCAC  ATGACACTCATACGTCGAATTCGCTTGTTCCAC | Biallelic |
| T0-11 | ATGACACTCATACGTCAAATTCGCTTGTTCCAC  ATGACACTCATACGTCGAATTCGCTTGTTCCAC | Biallelic |
| T0-12 | ATAACACTCATACGTCAAATTCGCTTGTTCCAC  ATGACACTCATACATCAAATTCGCTTGTTCCAC | Biallelic |
| T0-13 | ATGACACTCATACGTCAAATTCGCTTGTTCCAC  ATGACACTCATACGTCCAATTCGCTTGTTCCAC | Biallelic |
| T0-14 | ATGACACTCATACGTCAAATTCGCTTGTTCCAC  ATGACACTCATACGTCGAATTCGCTTGTTCCAC | Biallelic |
| T0-15 | ATGACACTCATACGTCAAATTCGCTTGTTCCAC  ATGACACTCATACGTCGAATTCGCTTGTTCCAC | Biallelic |
| T0-16 | ATGACACTCATACGTCAAATTCGCTTGTTCCAC  ATGACACTCATACGTCGAATTCGCTTGTTCCAC | Biallelic |
| T0-17 | ATGACACTCATACGTCAAATTCGCTTGTTCCAC  ATGACACTCATACGTCCAATTCGCTTGTTCCAC | Biallelic |
| T0-18 | ATGACACTCATACGTCAAATTCGCTTGTTCCAC  ATGACACTCATACGTCGAATTCGCTTGTTCCAC | Biallelic |
| T0-19 | ATGACACTCATACGTCAAATTCGCTTGTTCCAC  ATGACACTCATACGTCGAATTCGCTTGTTCCAC | Biallelic |
| T0-20 | ATGACACTCATACGTCAAATTCGCTTGTTCCAC  ATGACACTCATACGTCGAATTCGCTTGTTCCAC | Biallelic |
| T0-21 | ATAACACTCATACGTCAAATTCGCTTGTTCCAC  ATGACACTCATACGTCGAATTCGCTTGTTCCAC | Biallelic |
| T0-22 | ATGACACTCATACGTCAAATTCGCTTGTTCCAC  ATGACACTCATACGTCGAATTCGCTTGTTCCAC | Biallelic |
| T0-23 | ATGACACTCATACGTCAAATTCGCTTGTTCCAC  ATGACACTCATACGTCGAATTCGCTTGTTCCAC | Biallelic |
| T0-24 | ATGACACTCATACGTCAAATTCGCTTGTTCCAC  ATGACACTCATACGTCGAATTCGCTTGTTCCAC | Biallelic |
| T0-25 | ATGACACTCATACGTCAAATTCGCTTGTTCCAC  ATGACACTCATACGTCGAATTCGCTTGTTCCAC | Biallelic |
| T0-26 | ATGACACTCATACGTCAAATTCGCTTGTTCCAC  ATGACACTCATACGTCGAATTCGCTTGTTCCAC | Biallelic |
| T0-27 | ATGACACTCATACGTCAAATTCGCTTGTTCCAC  ATGACACTCATACGTCGAATTCGCTTGTTCCAC | Biallelic |
| T0-28 | ATGACACTCATACGTCAAATTCGCTTGTTCCAC  ATGACACTCATACGTCGAATTCGCTTGTTCCAC | Biallelic |
| T0-29 | Chimeric | Chimeric |
| T0-30 | ATCACACTCATACGTCAAATTCGCTTGTTCCAC  ATGACACTCATACATCAAATTCGCTTGTTCCAC | Biallelic |
| T0-31 | ATGACACTCATACGTCAAATTCGCTTGTTCCAC  ATGACACTCATACGTCGAATTCGCTTGTTCCAC | Biallelic |
| T0-32 | ATGACACTCATACGTCAAATTCGCTTGTTCCAC  ATGACACTCATACGTCGAATTCGCTTGTTCCAC | Biallelic |
| T0-33 | ATAACACTCATACGTCAAATTCGCTTGTTCCAC  ATGACACTCATACGTCGAATTCGCTTGTTCCAC | Biallelic |
| T0-34 | ATGACACTCATACGTCAAATTCGCTTGTTCCAC  ATGACACTCATACGTCGAATTCGCTTGTTCCAC | Biallelic |
| T0-35 | ATGACACTCATACGTCAAATTCGCTTGTTCCAC  ATGACACTCATACGTCGAATTCGCTTGTTCCAC | Biallelic |
| T0-36 | ATGACACTCATACATCAAATTCGCTTGTTCCAC  ATGACACTCATACGTCAAATTCGCTTGTTCCAC | Biallelic |
| T0-37 | ATGACACTCATACATCAAATTCGCTTGTTCCAC  ATGACACTCATACGTCGAATTCGCTTGTTCCAC | Biallelic |
| T0-38 | ATGACACTCATACATCAAATTCGCTTGTTCCAC  ATGACACTCATACGTCGAATTCGCTTGTTCCAC | Biallelic |
| T0-39 | ATGACACTCATACGTCAAATTCGCTTGTTCCAC  ATGACACTCATACGTCGAATTCGCTTGTTCCAC | Biallelic |
| T0-40 | ATGACACTCATACGTCAAATTCGCTTGTTCCAC  ATGACACTCATACGTCGAATTCGCTTGTTCCAC | Biallelic |
| T0-41 | ATGACACTCATACGTCAAATTCGCTTGTTCCAC  ATGACACTCATACGTCGAATTCGCTTGTTCCAC | Biallelic |
| T0-42 | ATGACACTCATACGTCAAATTCGCTTGTTCCAC  ATGACACTCATACGTCGAATTCGCTTGTTCCAC | Biallelic |
| T0-43 | ATGACACTCATACGTCAAATTCGCTTGTTCCAC  ATGACACTCATACGTCAAATTCGCTTGTTCCAC | Homozygous |
| T0-44 | ATGACACTCATACGTCAAATTCGCTTGTTCCAC  ATGACACTCATACGTCCAATTCGCTTGTTCCAC | Biallelic |
| T0-45 | ATGACACTCATACGTCAAATTCGCTTGTTCCAC  ATGACACTCATACGTCCAATTCGCTTGTTCCAC | Biallelic |
| T0-46 | ATGACACTCATACGTCAAATTCGCTTGTTCCAC  ATGACACTCATACGTCGAATTCGCTTGTTCCAC | Biallelic |
| T0-47 | ATGACACTCATACGTCAAATTCGCTTGTTCCAC  ATGACACTCATACGTCCAATTCGCTTGTTCCAC | Biallelic |
| T0-48 | Chimeric | Chimeric |
| T0-49 | ATGACACTCATACGTCAAATTCGCTTGTTCCAC  ATGACACTCATACGTCAAATTCGCTTGTTCCAC | Homozygous |
| T0-50 | Chimeric | Chimeric |
| T0-51 | Chimeric | Chimeric |
| T0-52 | ATGACACTCATACGTCAAATTCGCTTGTTCCAC  ATGACACTCATACGTCGAATTCGCTTGTTCCAC | Biallelic |
| T0-53 | ATGACACTCATACGTCAAATTCGCTTGTTCCAC  ATGACACTCATACGTCGAATTCGCTTGTTCCAC | Biallelic |
| T0-54 | A-----------------------GTCAAATTCGCTTGTTCCAC  ATGACACTCATACGTCAAATTCGCTTGTTCCAC | Biallelic |
| T0-55 | A-----------------------GTCAAATTCGCTTGTTCCAC  ATGACACTCATACGTCAAATTCGCTTGTTCCAC | Biallelic |
| T0-56 | ATGACACTCATACGTCAAATTCGCTTGTTCCAC  ATGACACTCATACGTCAAATTCGCTTGTTCCAC | Homozygous |
| T0-57 | ATGACACTCATACGTCAAATTCGCTTGTTCCAC  ATGACACTCATACGTCGAATTCGCTTGTTCCAC | Biallelic |
| T0-58 | Chimeric | Chimeric |
| T0-59 | ATGACACTCATACGTCAAATTCGCTTGTTCCAC  ATGACACTCATACGTCGAATTCGCTTGTTCCAC | Biallelic |
| T0-60 | ATGACACTCATACGTCAAATTCGCTTGTTCCAC  ATGACACTCATACGTCGAATTCGCTTGTTCCAC | Biallelic |
| T0-61 | ATGACACTCATACGTCAAATTCGCTTGTTCCAC  ATGACACTCATACGTCGAATTCGCTTGTTCCAC | Biallelic |
| T0-62 | Chimeric | Chimeric |
| T0-63 | ATGACACTCATACGTCAAATTCGCTTGTTCCAC  ATGACACTCATACGTCGAATTCGCTTGTTCCAC | Biallelic |
| T0-64 | ATGACACTCATACGTCAAATTCGCTTGTTCCAC  ATGACACTCATACGTCGAATTCGCTTGTTCCAC | Biallelic |
| T0-65 | ATGACACTCATACATCAAATTCGCTTGTTCCAC  ATGACACTCATACGTCGAATTCGCTTGTTCCAC | Biallelic |
| T0-66 | ATGACACTCATACGTCAAATTCGCTTGTTCCAC  ATGACACTCATACGTCGAATTCGCTTGTTCCAC | Biallelic |

Note: Nucleotide substitutions and small deletions are indicated in red.

**Table S2.** Potential off-target sites.

| Target site | Sequence (5’-3’) ^a^ | No. of mismatch | Target locus |
| --- | --- | --- | --- |
| On-target | GACGCATGAGTGTCATAGAA**AGG** | 0 | Fvb5:+28707054 |
| Off-target 1 | ccgGCAgGAGTGTCATAGAA**GGG** | 4 | Fvb6:+20951585 |
| Off-target 2 | GAtGCATGcaTGTCAgAGAA**GGG** | 4 | Fvb6:-19973548 |
| Off-target 3 | GAaGtATGAGTGTtcTAGAA**AGG** | 4 | Fvb3:-18022771 |

^a^ PAM motifs are written in bold and underlined; Mismatches are written in lowercase letters.

**Table S3.** Potential off-target analysis of the homozygous mutants.

|  | | AL1/AL1 | AL2/AL2 | AL3/AL3 | AL4/AL4 | AL5/AL5 | AL6/AL6 | AL7/AL7 |
| --- | --- | --- | --- | --- | --- | --- | --- | --- |
| Off-target 1 | NO | | NO | NO | NO | NO | NO | NO |
| Off-target 2 | NO | | NO | NO | NO | NO | NO | NO |
| Off-target 3 | NO | | NO | NO | NO | NO | NO | NO |

**Table S4.** Sequences of primers used to construct vectors and identify mutation events.

| Primer name | Primer sequence (5’-3’) | Application |
| --- | --- | --- |
| gRT02284  AtU6-29T02284  Pps-GGL  Pgs-GGR | GACGCATGAGTGTCATAGAAGTTTTAGAGCTAGAAAT  TTCTATGACACTCATGCGTCAATCTCTTAGTCGACT  TTCAGAGGTCTCTGGCGACTAGTATGGAATCGGCAGCAAAGG  AGCGTGGGTCTCGAAACACGCGTATCCATCCACTCCAAGCTC | sgRNA expression cassette construction |
| ProGFP f  ProGFP r | TGCATGCTGCAGGTCGACTCT  GCTTGCATGCCTGCAGGTCAC | GFP expression cassette construction |
| uORF5 f  uORF5 r | CTGTACATGATTACGCCAACC  CTCTTCTTGTCGTCCACCAC | Amplifying the uORF target site |
| Primer set1 f  Primer set1 r | ACGACGGCAACTACAAGACCC  TCACGAACTCCAGCAGGACCA | Detecting T-DNA insertion |
| Primer set2 f  Primer set2 r | TCGCCATTGGGACTAACTCTG  TTCTCCTCGAAGAGCTGGTT | Detecting T-DNA insertion |
