# Supplementary material for: Fine-tuning sugar content in strawberry
Source: Genome Biol. 2020 Sep 3;21:230. doi: 10.1186/s13059-020-02146-5 (PMC7470447; doi:10.1186/s13059-020-02146-5)
Supplement: Supplementary file 2 — Additional file 2. Sequences S1. 5′ untranslated region (UTR) of the gene FvebZIPs1.1. Sequences S2. Coding sequences of FvebZIPs1.1. [file 13059_2020_2146_MOESM2_ESM.docx]

**Sequences S1.** 5’ untranslated region (UTR) of the gene *FvebZIPs1.1*. The conserved SC-uORF is colored in red.

**>5’ untranslated region (UTR) of the gene *FvebZIPs1.1***

ATAAAACCAACCCTCCATCCCCATTGTTAACCTTCCCTTATTCTTCTTTGCTTTTCACATAGCCTCCCTCTGTTTCTCCGCCTCAGCTTTCAGACAAAGTTGCTCTAGTTGCCTTGTCTTTCTCTCCCTCCAGAGATCTCTCTCTGTTTGTTTGTTTTCGGGGTTTGTTGAAATTTCATTTCGATTTTTTCTGATTTTGGGGATCTGGGATTTCTAGGGGTTCTTTGTAACGTAAGCCCAATGTGTTCAGAAGCCTTTCTATGACACTCATGCGTCGAATTCGCTTGTTCCACTCTTTCTCCGTCGCCTTCCTGTACTGGTTCTACGTTTTCTCATGAACTAATCCACCCAGATTCCTCATTTCAATTTCTCAATTTTCGAAAACCCCCAATTTGAAACCCTAATTTTTTCAATTTCTTAATTTATTGAACCGATTCTTCCTGAG

**Sequences S2.** Complete coding sequences of *FvebZIPs1.1*.

**>Coding sequences of *FvebZIPs1.1***

ATGTCTTCGGTTCAGCGCCAATCGAGCTCCGGCTCCGACGGATGCGTGGTGGACGACAAGAAGAGGAAGAAGATGGAGTCCAACCGCGAATCGGCGCGGCGATCAAGGATGAAGAAGCAGAAGCACGTGGAGGAGCTGACGGTGGAGATCACCCGGCTCCAGATCTCCAACAACCAGCTCCGGCAGAGCATCGACGCCAAGGATAAGGCCTACAGCGAGTTGGAGGCCTGGAACAATGTCATGAGGGCTCAGGAGAAGGAGCTCACGGATCGGCTGCGCTACTTGGAGTCGATTGAGCAGACATTTGTGGAGTTCAGCGGCGGATTCGATGAGAACATGAATGGCATGGCTGAGATGCATGACACAATGCTCAATCCGTGGCAGATTCCTTACTCTACTCAGCCCATCACGGCTTCCTCTGCCGATATGTTTCTCGATTGGCCATAA
