# Supplementary material for: Fine-tuning sugar content in strawberry
Source: Genome Biol. 2020 Sep 3;21:230. doi: 10.1186/s13059-020-02146-5 (PMC7470447; doi:10.1186/s13059-020-02146-5)
Supplement: Supplementary file 3 — Additional file 3. Review history. [file 13059_2020_2146_MOESM3_ESM.docx]

Review History

**First round of review**

**Reviewer 1**

**Were you able to assess all statistics in the manuscript, including the appropriateness of statistical tests used?**

Yes: The statistics in this manuscript is qualified

**Were you able to directly test the methods?**

Yes

**Comments to author:**

This manuscript presented a work on base editing of highly conserved upstream ORF of FvebZIPs1.1 in strawberry. A version of base editor A3A-PBE, a wide editing window tool, was used to generate a series of mutants with T-DNA free. And, higher sugar contents mutants were verified at homozygous T1 mutant lines and hence they were verified heritable to next generation generated from asexual reproduction. The key merit of this paper is that it is a completed work on trait improvement using base editing in strawberry. In my opinion, the experimental design, experimental procedures, data analysis, and interpretation of the results are clear, complete, and the logic. To my knowledge, it is the first work reported in this fruit crop by using this tool. This work may attract broad research interests from developing plant genome editing tools, application of genetic improvement on fruits and other crops.
I here just list 2 minor concerns, please discuss it and/or provide some extended data if author have had.
i)     The target gene is uORF of FvebZIPs1.1, a transcription factor regulator, sense mutations had been made to produce a series of mutations. Existing data had proved that the target trait, the sugar content, had a significant impact thanks to mutations, and there was no side effects on plant growth and morphology. It would be more perfect if there were data on the main quality traits other than the target trait, the sugar content, of the created mutants. It should be pointed out that this data is not necessary, it is better if there was, since the objective of this study is quality improvement.
ii)     The author's group and other research groups have published papers on off-target mutation by using CBE. The previous data in other plant species indicated that the CBE might cause some level or substantial off-target mutations in recipient plants. We didn't see off-target data and analysis in the presented manuscript. I believe other readers will be very interested on this point as I do. If there were such data, please show it at revision. If no, could you please discuss some on this point?
In summary, I suggest this paper could be acceptable for publication on Genome Biology after minor revision.

**Reviewer 2**

**Were you able to assess all statistics in the manuscript, including the appropriateness of statistical tests used?**

No

**Were you able to directly test the methods?**

No

**Comments to author:**

This study applied uORF editing to presumably improve protein translation in a diploid strawberry for altering sugar content. As the authors stated, it is a first report of base editing in strawberry. The study also provides a useful gene editing method to improve fruit sugar content. However, I have concerns about the establishment of a strong genotype-phenotype relationship in this case study.

Major points.
1)     What is the rationale that a dual luciferase assay is valid assay for studying an uOFR? The 35S promoter is a very strong promoter which will pretty much overwrite all other cis elements' effects and limit the possibility of seeing even higher protein accumulation on top of the high amount of mRNA. Also, the uOFR's regulation is depending on its encoded peptide here and its down-stream gene (e.g. bZIP1). By replacing bZIP1 with a luciferase gene will completely take this regulatory mechanism out of the context; yet the authors wanted to study such relationship. A more convincing assay may use a genomic promoter and genomic gene fusion (with a LUC gene) so that endogenous regulatory mechanisms are preserved. However, it is far more convincing to do a direct target protein quantification (see below).
2)     It remains unclear the increased sugar contents were due to enhanced protein translation or due to reduced transcription of FvebZIPs1.1 in strawberry, as both would contribute to opposite phenotypes. I am puzzled with the genotype to phenotype linkage in this case. Even if the dual luciferase assay truly suggests enhanced protein translation of luciferase, the results may not be applicable to the FvebZIPs1.1 protein as I questioned above. The regulation mechanism of FvebZIPs1.1 in strawberry was not elucidated before. We cannot assume FvebZIPs1.1 will be just the same as one similar gene in Arabidopsis and tomato, especially there are multiple homologous bZIP genes in strawberry and they may have varied regulatory modes. Consequently, I feel key evidence for enhanced protein translation of FvebZIPs1.1 leading to enhanced sugar contents needs to be firmly established, given the fact the mRNA of FvebZIPs1.1 is down regulated in the mutants. Previous studies have established higher sucrose as well as other sugar contents with bZIP1 overexpression (e.g. Plant Biotechnology Journal (2016) 14, pp. 1116-1126). However, many mutant lines in this current strawberry study actually showed lower gene expression, which would indicate lower sugar content, an opposite to the authors' conclusion. Hence, it will be critical to show the significantly enhanced protein translation of FvebZIPs1.1 in these mutants, which should be readily detected with any protein detection and quantification methods. For example, either western blot or mass spectrometry can be applied to quantify the amount of FvebZIPs1.1 protein in mutant lines and the wild type plants. Without showing the mutants have higher FvebZIPs1.1 protein level, this reviewer feels the genotype-phenotype causal relationship has not been firmly established in this study.

Minor points:

1)     Line 106: Remove "it's".
2)     Line 195-200: Why sucrose content was not altered in the mutants, yet fructose and glucose contents were significantly higher in the mutants? Do authors suggest that a steady level of sucrose needs to be maintained and the excess amount of sucrose will be rapidly converted to fructose and glucose by the invertase? If so, I suggest the authors propose a mechanism with citing relevant references.
3)     Figure 1a: The DNA direction was wrongly indicated. It should be 3' to 5' base on the PAM and protospacer sequence

**Authors Response**

**Point-by-point responses to the reviewers’ comments:**

Comment

*Response*

Reviewer #1: ===This manuscript presented a work on base editing of highly conserved upstream ORF of FvebZIPs1.1 in strawberry. A version of base editor A3A-PBE, a wide editing window tool, was used to generate a series of mutants with T-DNA free. And, higher sugar contents mutants were verified at homozygous T1 mutant lines and hence they were verified heritable to next generation generated from asexual reproduction. The key merit of this paper is that it is a completed work on trait improvement using base editing in strawberry. In my opinion, the experimental design, experimental procedures, data analysis, and interpretation of the results are clear, complete, and the logic. To my knowledge, it is the first work reported in this fruit crop by using this tool. This work may attract broad research interests from developing plant genome editing tools, application of genetic improvement on fruits and other crops. I here just list 2 minor concerns, please discuss it and/or provide some extended data if author have had.

i)	The target gene is uORF of FvebZIPs1.1, a transcription factor regulator, sense mutations had been made to produce a series of mutations. Existing data had proved that the target trait, the sugar content, had a significant impact thanks to mutations, and there was no side effects on plant growth and morphology. It would be more perfect if there were data on the main quality traits other than the target trait, the sugar content, of the created mutants. It should be pointed out that this data is not necessary, it is better if there was, since the objective of this study is quality improvement.

*Response: Thanks for the suggestion. We have added the Citric Acid and* *Malic Acid data into Additional file 1: Figure S6 in the reviript. Organic acids (citric acid and malic acid) are key components impacting fruit flavor. The analyses showed that the citric acid was slightly lower in homozygous mutants for AL5 and AL6 than that in WT, but malic acid contents in the seven homozygous mutants were not affected (L217-L221).*

ii)    The author's group and other research groups have published papers on off-target mutation by using CBE. The previous data in other plant species indicated that the CBE might cause some level or substantial off-target mutations in recipient plants. We didn't see off-target data and analysis in the presented manuscript. I believe other readers will be very interested on this point as I do. If there were such data, please show it at revision. If no, could you please discuss some on this point?

*Response: Thanks for the suggestion. We have checked whether off-target mutations were induced in the mutants and added the date into Additional file 1: Tables S2 and S3 in the revised manuscript. We searched the genomic sequence for all target sites that contained sequences with up to a 4-nt mismatch and sequenced these sites in the homozygous mutants. We found no off-target mutations in any of the homozygous mutants (L192-L196). We have discussed sgRNA-independent off-target mutations in discussion in the revised manuscript (L299-L303). Although we found no mutations in potential off-target sites, sgRNA-independent off-target mutations may exist. Most recently, new cytosine base editors, A3Bctd-VHM-BE3 and A3Bctd-KKR-BE3, exhibit high specificity and precision in plants, which can eliminate sgRNA-independent DNA off-target edits [58].*

In summary, I suggest this paper could be acceptable for publication on Genome Biology after minor revision.

Reviewer #2: This study applied uORF editing to presumably improve protein translation in a diploid strawberry for altering sugar content. As the authors stated, it is a first report of base editing in strawberry. The study also provides a useful gene editing method to improve fruit sugar content. However, I have concerns about the establishment of a strong genotype-phenotype relationship in this case study.Major points.

1)    What is the rationale that a dual luciferase assay is valid assay for studying an uOFR? The 35S promoter is a very strong promoter which will pretty much overwrite all other cis elements' effects and limit the possibility of seeing even higher protein accumulation on top of the high amount of mRNA. Also, the uOFR's regulation is depending on its encoded peptide here and its down-stream gene (e.g. bZIP1). By replacing bZIP1 with a luciferase gene will completely take this regulatory mechanism out of the context; yet the authors wanted to study such relationship. A more convincing assay may use a genomic promoter and genomic gene fusion (with a LUC gene) so that endogenous regulatory mechanisms are preserved. However, it is far more convincing to do a direct target protein quantification (see below).

*Response: Thanks for the comments. We agree that the dual luciferase assay may not fully demonstrate endogenous regulatory mechanisms and the uORF function is better to be confirmed under the native promoter and gene context. But this is a routine transient method used for studying the effect of uORF on translational efficiency in vitro. In our previous studies, we have proved the effects of uORFs with different genes using this Pro35S promoter:uROF::LUC construct, and higher protein accumulation was observed when the uORF was mutated. [e.g. Nature (2017) vol. 545, pp. 491-493; Nature Biotechnology (2018) vol. 36, pp. 894-898]. Therefore, it can be used to prove the uORF effects as an in vitro experiment.*

2)    It remains unclear the increased sugar contents were due to enhanced protein translation or due to reduced transcription of FvebZIPs1.1 in strawberry, as both would contribute to opposite phenotypes. I am puzzled with the genotype to phenotype linkage in this case. Even if the dual luciferase assay truly suggests enhanced protein translation of luciferase, the results may not be applicable to the FvebZIPs1.1 protein as I questioned above. The regulation mechanism of FvebZIPs1.1 in strawberry was not elucidated before. We cannot assume FvebZIPs1.1 will be just the same as one similar gene in Arabidopsis and tomato, especially there are multiple homologous bZIP genes in strawberry and they may have varied regulatory modes. Consequently, I feel key evidence for enhanced protein translation of FvebZIPs1.1 leading to enhanced sugar contents needs to be firmly established, given the fact the mRNA of FvebZIPs1.1 is down regulated in the mutants. Previous studies have established higher sucrose as well as other sugar contents with bZIP1 overexpression (e.g. Plant Biotechnology Journal (2016) 14, pp. 1116-1126). However, many mutant lines in this current strawberry study actually showed lower gene expression, which would indicate lower sugar content, an opposite to the authors' conclusion. Hence, it will be critical to show the significantly enhanced protein translation of FvebZIPs1.1 in these mutants, which should be readily detected with any protein detection and quantification methods. For example, either western blot or mass spectrometry can be applied to quantify the amount of FvebZIPs1.1 protein in mutant lines and the wild type plants. Without showing the mutants have higher FvebZIPs1.1 protein level, this reviewer feels the genotype-phenotype causal relationship has not been firmly established in this study.

*Response: Thanks for the comments. Sucrose-induced repression of translation (SIRT) of S1-group bZIP (having the conserved uORF in 5’UTR) genes by the conserved uORF has been well established [e.g. The Plant Cell (2004) vol. 16, pp. 1717-1729; Plant Physiology (2009) vol. 150, pp. 1356-1367]. The functions of S1-group bZIP genes have also been clearly demonstrated [e.g. The Plant Cell (2011) vol. 23, pp. 381-395; The Plant Cell (2018) vol. 30, pp. 495-509]. The uORF is highly conserved in many dicotyledonous and monocotyledonous plants, such as Arabidopsis (AtbZIP1, AtbZIP2, AtbZIP11, AtbZIP44, AtbZIP53), tobacco (Nttbz17, NttbzF), maize (ZmmLIP15, ZmOCSBF-1), snapdragon (AmbZIP910), soybean (GmATB2), parsley (Pccprf6), pepper (CcPPI1) and tomato (SlbZIP1). The sucrose-induced repression of translation mechanism has also been demonstrated in Arabidopsis, tobacco, tomato and even gymnosperms. These findings suggest the a conserved bZIP gene function and regulation mechanism may be conxerved in plants.*

*Low energy stress activated the Arabidopsis thaliana group S1 bZIP transcription factors AtbZIP1 and AtbZIP53 by both transcriptional and posttranscriptional mechanisms [The Plant Cell (2011) vol. 23, pp. 381-395]. Deregulation of sucrose-controlled translation of tobacco gene TBZ17 (homologues of AtbZIP53) resulted in sucrose accumulation in tobacco leaves [PLoS one (2012) vol. 3, Issue 3]. Overexpression of SlbZIP1 and SlbZIP1 (homologues of AtbZIP53) under the control of the fruit-specific E8 promoter, without the SIRT responsive uORFs, resulted in higher sugar content [Plant Biotechnology Journal (2016) vol. 14, pp. 1116-1126]. There are four strawberry bZIP genes that contain the conserved uORF in 5’UTR. Phylogenetic analysis revealed that only the FvebZIPs1.1 was the homologues of AtbZIP53 (Figure 1b), TBZ17 and SlbZIP1 (Additional file 1 Figure S1). Moreover, only the conserved uORF of FvebZIPs1.1 encoded a short peptide with 25 amino acids as AtbZIP53, TBZ17 and SlbZIP1 (Figure 1d), but the conserved uORFs of the other three strawberry genes encoded long peptides with 41 amino acids (Figure 1d).*

*The regulation mechanism of FabZIPs1.1 (the same gene of FvebZIPs1.1) was also elucidated before in culture octoploid strawberry [Scientia Horticulturae (2020) Volume 259 https://doi.org/10.1016/j.scienta.2019.108850]. FabZIPs1.1 was transcriptionally repressed by both blue and red light in strawberry fruits. It was extensively expressed in both vegetative and reproductive organs. The transcriptional level of FabZIPs1.1 was not affected by sucrose under in vitro conditions. FabZIPs1.1 not only exhibited sucrose induced repression of translation property, but also possessed glucose-induced translational repression under light conditions tested in stable transgenic Arabidopsis lines. Transient overexpression of FabZIPs1.1 lacking the leader sequence under the control of CaMV 35S promoter in strawberry fruits could enhance sucrose accumulation. We have cited this paper in the revised manuscript (L115, L511-L514).*

*Editing plant uORFs provides a generalizable and efficient method for manipulating the translation of mRNA. In the AtBRI1 uORF mutants, AtBRI1 protein was upregulated (quantified by Western blot). Editing uORFs of AtVTC2, LsGGP1 and LsGGP2 significantly increased ascorbic acid content in Arabidopsis and lettuce [Nature Biotechnology (2018) vol. 36, pp. 894-898]. In this study, we created FvebZIPs1.1 uORF mutants (diploid strawberry) by base editing and obtained transgene-free homozygous mutants in T1 generation. Sugar contents of the homozygous T1 mutant lines were significantly higher than that of wild type. In the mutants, the FvebZIPs1.1 was driven by the genomic promoter, therefore, those endogenous regulatory mechanisms are preserved. The reduced transcription of FvebZIPs1.1 in mutants might be caused by the increased sugar content, as reported in Arabidopsis that sucrose and glucose repressed AtbZIP1 and AtbZIP53 transcript accumulation [The Plant Cell (2011) vol. 23, pp. 381-395]. Because previous studies had established higher sugar contents with bZIP overexpression in tobacco, tomato and strawberry, we hypothesized that the increased sugar content might not due to the reduced transcription of FvebZIPs1.1 in strawberry.*

*As to the protein quantification experiment, we are very sorry that we cannot get the antibody to quantify the amount of FvebZIPs1.1 protein by Western blot. We also cannot quantify the amount of FvebZIPs1.1 protein by mass spectrometry because of the low protein abundance of this transcription factor in mature strawberry fruits which may lead to low accuracy.*

Minor points:

1)	Line 106: Remove "it's".

*Response: Thanks for the correction. We have removed "it's" in the revised manuscript (Line 106).*

2)    Line 195-200: Why sucrose content was not altered in the mutants, yet fructose and glucose contents were significantly higher in the mutants? Do authors suggest that a steady level of sucrose needs to be maintained and the excess amount of sucrose will be rapidly converted to fructose and glucose by the invertase? If so, I suggest the authors propose a mechanism with citing relevant references.

*Response: Thanks for the suggestion. We have proposed a mechanism by citing relevant references in the revised manuscript (L205-L210, L519-L521). The significantly higher fructose and glucose, but not sucrose contents in mutants may be due to the high and constant invertase activity in fruits. The major function of the high and constant invertase activity in fruit is to maintain high cellular hexose concentrations. The hydrolysis of sucrose in the vacuole and in the intercellular space allows more efficient storage of sugar in these compartments.*

3)    Figure 1a: The DNA direction was wrongly indicated. It should be 3' to 5' base on the PAM and protospacer sequence.

*Response: Thanks for pointing out the error. We have corrected in the revised Figure 2a.*

**Second round of review**

**Reviewer 1**

I think the revised manuscript and the provided additional data have well addressed the concerns raised during the first round of review. I suggest the current version could be acceptable for publication on Genome Biology.
